# Enhanced hydrogenation activity and diastereomeric interactions of methyl pyruvate co-adsorbed with *R*-1-(1-naphthyl)ethylamine on Pd(111)

**DOI:** 10.1038/ncomms12380

**Published:** 2016-08-04

**Authors:** Mausumi Mahapatra, Luke Burkholder, Michael Garvey, Yun Bai, Dilano K. Saldin, Wilfred T. Tysoe

**Affiliations:** 1 Department of Chemistry and Biochemistry, University of Wisconsin-Milwaukee, Milwaukee, Wisconsin 53211, USA; 2 Applied Research Institute, University of Illinois at Urbana-Champaign, Champaign, Illinois 61820, USA; 3 Department of Physics, University of Wisconsin-Milwaukee, Milwaukee, Wisconsin 53211, USA

## Abstract

Unmodified racemic sites on heterogeneous chiral catalysts reduce their overall enantioselectivity, but this effect is mitigated in the Orito reaction (methyl pyruvate (MP) hydrogenation to methyl lactate) by an increased hydrogenation reactivity. Here, this effect is explored on a *R*-1-(1-naphthyl)ethylamine (NEA)-modified Pd(111) model catalyst where temperature-programmed desorption experiments reveal that NEA accelerates the rates of both MP hydrogenation and H/D exchange. NEA+MP docking complexes are imaged using scanning tunnelling microscopy supplemented by density functional theory calculations to allow the most stable docking complexes to be identified. The results show that diastereomeric interactions between NEA and MP occur predominantly by binding of the C=C of the *enol* tautomer of MP to the surface, while simultaneously optimizing C=O····H_2_N hydrogen-bonding interactions. The combination of chiral-NEA driven diastereomeric docking with a tautomeric preference enhances the hydrogenation activity since C=C bonds hydrogenate more easily than C=O bonds thus providing a rationale for the catalytic observations.

## Abstract (web summary)

Achiral sites on a catalyst can result in formation of racemic product, though this can be avoided where the chiral sites lead to enhanced reaction rates. Here, the authors report that for the hydrogenation of methyl pyruvate the chiral modifier enhances the hydrogenation reactivity by favouring the more reactive enol tautomer.

## Graphical abstract

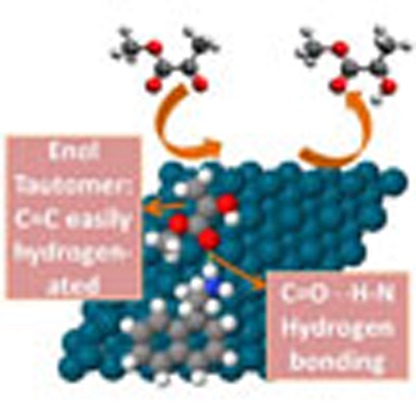

Pharmaceuticals and agrochemicals must be produced in an enantiopure form^1^, and are most often synthesized using organometallic catalysts with a chiral stereo-directing ligand at the reaction centre^2^,^3^,^4^,^5^; there are currently many examples of highly enantioselective, homogeneous-phase chiral catalysts^6^. However, this approach requires the stringent removal of the heavy metals from the product mixture so that the use of heterogeneous-phase chiral catalysts would therefore be beneficial. Organometallic catalysts can be heterogenized by anchoring them to a support, but such tethered catalysts can suffer from problems such as leaching or reduced enantioselectivity^7^,^8^,^9^. An alternative approach is to implement a strategy analogous to that exploited in homogeneous phase, by directly modifying the catalyst surface using a chiral ligand. Here, the chiral modifiers can be classified as those that act as templates or one-to-one modifiers^10^. Templates function by several chiral adsorbates acting in concert to provide enantioselective reaction sites, while one-to-one modifiers operate via a direct diastereomeric interaction between the modifier and prochiral reactant^11^. However, while all the reaction centres of an organometallic compound are influenced by the chiral ligand, it is a major challenge to modify all reaction sites on an extended surface. As unmodified sites will form racemic products, this reduces the overall enantioselectivity. This could conceivably be addressed by forming an extended template array which exposes only enantioselective pockets, but this has not, as yet, been achieved. Nonetheless, heterogeneous chiral catalysts have been synthesized, in particular for the enantioselective hydrogenation of α-ketoesters. For example, the CH_3_–C=O bond in methyl pyruvate (MP) can be enantioselectively hydrogenated to methyl lactate over cinchona-modified catalysts in the so-called Orito reaction where cinchona operates as a one-to-one modifier^12^,^13^,^14^,^15^,^16^,^17^,^18^,^19^,^20^,^21^,^22^,^23^,^24^. In this case, it has been found that hydrogenation rates are substantially increased at chirally modified sites compared with the unmodified catalyst^17^,^22^,^24^,^25^,^26^,^27^, thereby enhancing the effect of the diastereomeric interaction to produce enantiomeric excesses (ee) approaching 100%. This effect provides a potential solution to this central problem in designing heterogeneous chiral catalysts, but the origin of this rate enhancement is not well understood.

To explore the diastereomeric interactions between MP and chiral modifiers, and to investigate the origins of the enhanced hydrogenation activity, experiments are performed on a model Pd(111) single-crystal catalyst modified by *R*-1-(1-naphthyl)ethylamine (NEA) since the cinchona alkaloids are difficult to introduce into vacuum. NEA shares the key functional aspects of cinchonidine and has been used as a model chiral modifier^28^,^29^,^30^,^31^,^32^, where both endo and exo adsorbate conformers have been identified^31^. NEA has also been found to be randomly distributed on surfaces thus implying that NEA should act as a one-to-one modifier to provide a chiral reaction site^30^,^31^. The chemistry of the simplest *α*-ketoester, MP has been explored on several transition-metal surfaces^33^,^34^,^35^,^36^,^37^,^38^ where it exists as both the *enol* and *keto* tautomer (depicted in Supplementary Fig. 4)^36^,^38^ and the energetics on Pd(111) have been discussed in detail^37^. However, MP tends to agglomerate on platinum, the most commonly used catalyst for the enantioselective hydrogenation of MP (ref. 39), making it difficult to identify docking complexes between MP and NEA using scanning tunnelling microscopy (STM) on this surface. MP interacts to a lesser extent on Pd(111), thereby optimizing the possibility of observing interactions between MP and NEA.

The results show that complexes predominantly form with the *enol* tautomer of MP. The most stable diastereomeric interactions between MP and NEA occur when the C=C bond of the *enol* tautomer binds to the most stable Pd bridge site, while simultaneously optimizing the C=O····H_2_N hydrogen-bonding interactions between NEA and MP. Since C=C double bonds hydrogenate significantly more easily than C=O bonds^40^, this provides a plausible explanation for the enhanced hydrogenation activity found on chirally modified catalysts.

## Results

### Temperature-programmed desorption experiments

**Figure 1:**
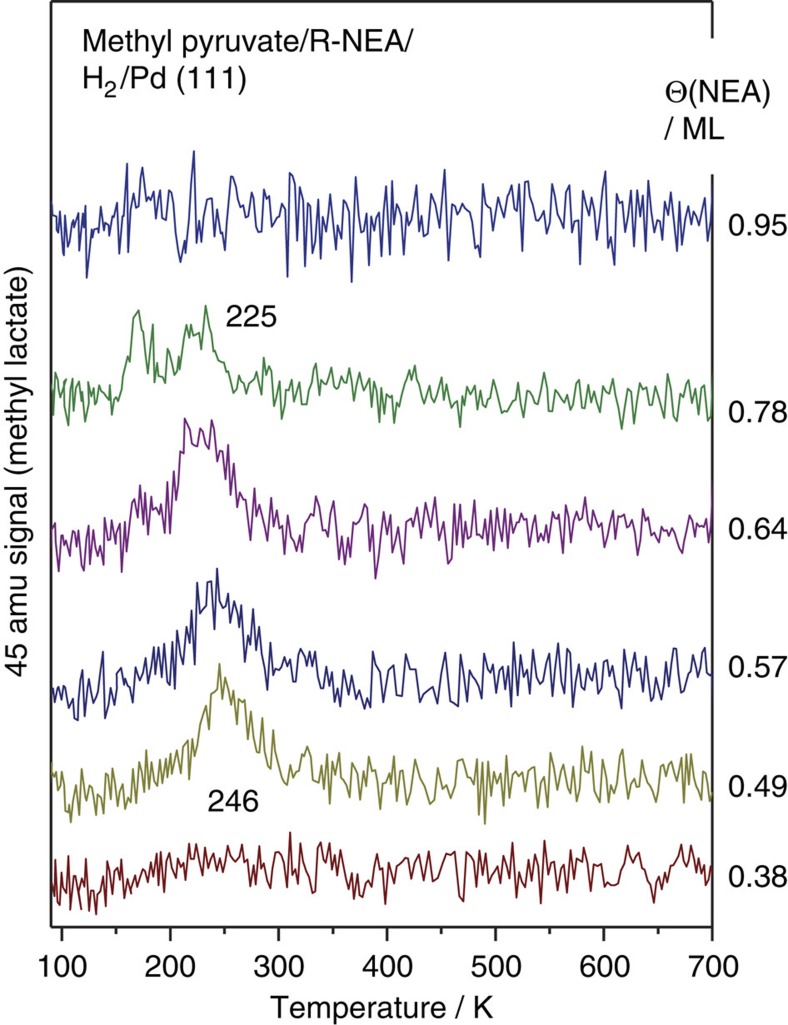
TPD profiles showing the effect of NEA co-adsorption on MP hydrogenation on Pd(111). The traces show the 45 AMU (methyl lactate) TPD profiles collected following the adsorption of 3 L (1 L (Langmuir)=1 × 10^−6^ Torr·s) of MP on a Pd(111) surface precovered with NEA and saturated with hydrogen, all dosed at a sample temperature of ∼90 K, as a function of NEA coverage. The NEA coverages are measured by CO site blocking and the values are displayed adjacent to the corresponding desorption profile.

To understand the enhancement in hydrogenation activity, we first explore the hydrogenation of MP on NEA-modified Pd(111) using temperature-programmed desorption (TPD) to establish that a Pd(111) single crystal adequately reproduces this enhancement effect. A Pd(111) surface was dosed with various coverages of NEA, where the coverages were determined by carbon monoxide site blocking as described previously^41^. The surface was then saturated with atomic hydrogen (3 l (1 L (Langmuir)=1 × 10^−6^ Torr·s) exposure), and finally saturated with MP. The resulting 45 AMU (the most intense signal of methyl lactate, assigned to the CH_3_–CH–OH^+^ mass spectrometer ionizer fragment) TPD profiles are displayed in Fig. 1, as a function of NEA coverage. The most intense mass spectrometer fragment of MP is at 43 AMU (from the CH_3_–C=O^+^ fragment), with a small (∼2% relative intensity) signal at 45 AMU mass, giving rise to the multilayer MP feature at ∼170 K (ref. 37), which is evident primarily at an NEA coverage of 0.78 monolayers. A clear methyl lactate desorption peak is evident depending on the *R*-NEA coverage, which is centred at ∼246 K for a NEA coverage of 0.49 monolayers, decreasing in temperature to ∼225 K at higher NEA coverages. The assignment of this feature to methyl lactate was confirmed by measuring the signals at other masses. The methyl lactate yield is below the detection limit for NEA coverages of 0.38 monolayers and lower (data not shown), but the methyl lactate yield increases with increasing NEA coverage demonstrating that MP hydrogenation is enhanced by the presence of NEA. The hydrogenation yield decreases to zero once again at the highest NEA coverage as MP adsorption is blocked. The proximity of the methyl lactate desorption temperature (from ∼225 to 246 K) to that for ethylene hydrogenation on Pd(111) (∼250 K, Supplementary Fig. 1, ref. 42) is consistent with it forming by hydrogenation of a C=C double bond, indicating that the methyl lactate forms by hydrogenation of the *enol* tautomer of MP.

**Figure 2:**
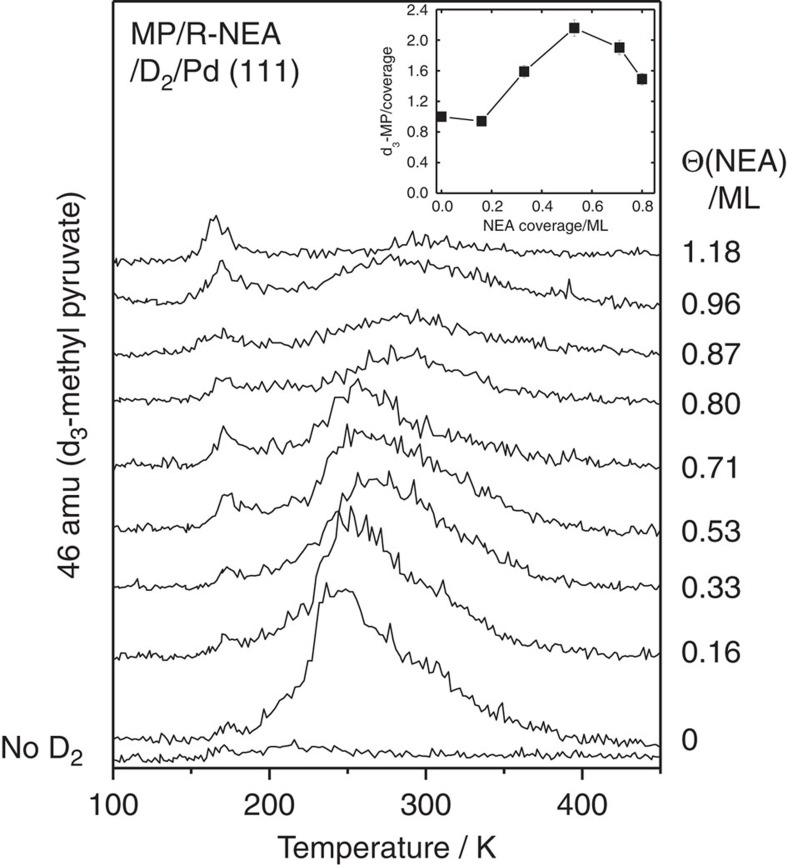
TPD profiles showing the effect of NEA co-adsorption on hydrogen/deuterium exchange on Pd(111). The traces show the 46 AMU (d_3_-MP) TPD profiles collected following the adsorption of 3 L of MP on a Pd(111) surface precovered with NEA and saturated with hydrogen, all dosed at a sample temperature of ∼90 K, as a function of NEA coverage. The NEA coverages are measured by CO site blocking and the values are displayed adjacent to the corresponding desorption profile.

To further explore the influence of *keto*-*enol* tautomerization and, since H/D exchange occurs for ethylene on group VIII transition metals^43^ including Pd(111) (Supplementary Fig. 2, Supplementary Table 1), TPD experiments were carried out for MP adsorbed on NEA-precovered Pd(111) that had been saturated with deuterium. The resulting 46 AMU desorption profiles are shown in Fig. 2 for various NEA coverages. The peak desorption temperature and shape indicate that the feature is due to MP desorption so that the peak is assigned to d_3_-MP (from the CD_3_–C=O^+^ fragment). The results indicate that MP has undergone substantial H/D exchange, again providing clear evidence of the participation of the *enol* form of MP in the reaction. No 46 AMU signal is detected in the absence of deuterium (bottom profile, Fig. 2), but H/D exchange occurs even without NEA (Θ(NEA)=0 monolayers). The inset to Fig. 2 shows the integrated area of the 46 AMU signals ratioed to the relative MP coverage, which decreases due to site blocking by NEA, and was measured from the integrated intensity of 43 AMU TPD profiles collected in the same experiment. This ratio increases over the same NEA coverage range as that at which MP hydrogenation is observed (Fig. 1).

### NEA Adsorption Site on Pd(111)

Previous density functional theory (DFT) calculations have indicated that the naphthyl ring of NEA is located on the so-called dibridge[7] site (Supplementary Fig. 3)^32^,^44^, while calculations on Pt(111) have suggested that it adsorbs on the dibridge[6] site (Supplementary Fig. 3)^39^,^45^. The dibridge[7] site on Pd(111) was previously identified by first calculating the most stable naphthalene adsorption site (dibridge[7]^44^) and then by adding an ethylamine group to calculate the final structure. Since the separation between NEA and MP in the docking complex is likely to significantly influence the nature of the diastereomeric interactions, we recalculated the most stable structure of NEA on Pd(111) adsorbed at various sites and found that the dibridge[6] site was, in fact, also more stable on Pd(111) than adsorption on the dibridge[7] site, where the exo conformer is more stable by 20 kJ mol^−1^ and the endo structure by 11 kJ mol^−1^ (Supplementary Discussion). To unequivocally establish the correct NEA adsorption site, we measured the low-energy electron diffraction (LEED) intensity versus beam voltage (I/V) curves of NEA on Pd(111) (Supplementary Fig.18)^46^. NEA on the dibridge[6] site was found to have the lowest Pendry R-factor (0.15 for the dibridge[6] site compared with 0.23 for the dibridge[7] site; Supplementary Table 13). This reveals that the binding of NEA to the surface is not only controlled by the preferred naphthyl adsorption site but is also strongly influenced by the binding of the amine lone pair to a palladium atop site, which then shifts the ring from a dibridge[7] to a dibridge[6] site.

### Scanning tunnelling microscopy of docking complexes

**Figure 3:**
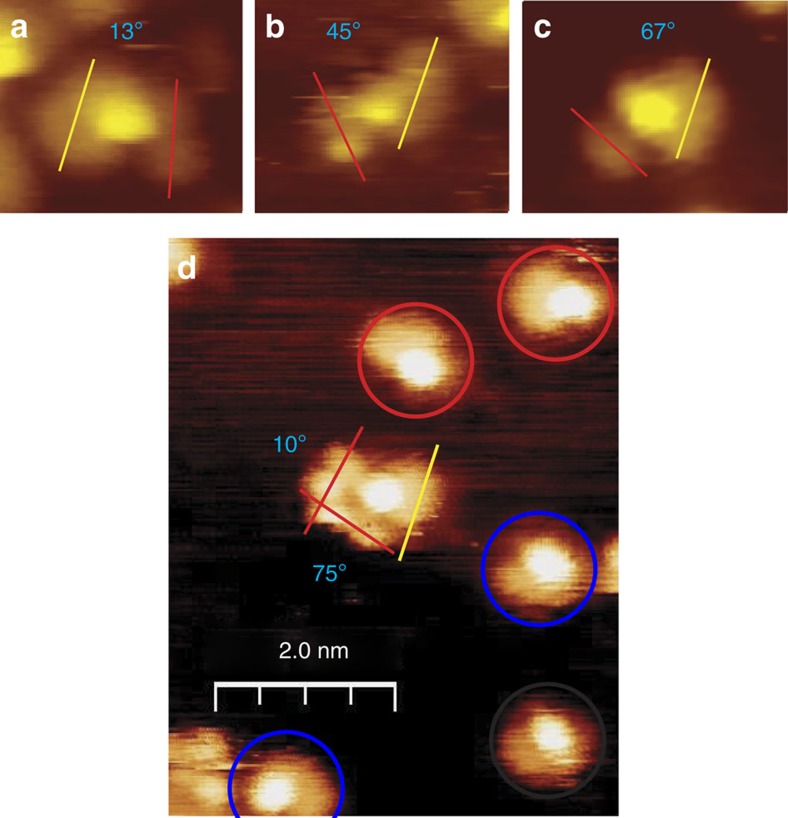
Example images of docking complexes formed between MP and NEA on Pd(111). The figure shows high-resolution STM images of the docking complexes identified from co-adsorbed NEA and MP on Pd(111) at 120 K. In most cases, a single MP molecule coordinates to NEA. Examples of such docking complexes are shown in **a**–**c** and are characterized based on the angles between the long axes of the naphthyl group (indicated as yellow lines) and the MP molecule (indicated as red lines). The examples shown, which appear most often, have dihedral angles of ∼13° (**a**), 45° **(b**) and 67° (**c**). In rare cases, shown in **d**, which displays a wider scan area, two MP molecules can coordinate to a NEA molecule. This figure also illustrates that the docking complexes are well separated from isolated NEA molecules, which are highlighted in red (exo conformer) and blue (endo conformer).

The docking complexes formed between prochiral MP and the chiral modifier, NEA are explored by imaging them using STM. First-principles DFT calculations and STM image simulations are used to identify the nature of the docking complexes and to compare the theoretically predicted and experimental distributions of the various types of docking complex, similar to previous approaches to understand chirality transfer on platinum substrates^35^,^39^,^47^,^48^,^49^. STM images of co-adsorbed NEA and MP on Pd(111) were collected at a sample temperature of ∼120 K by first exposing the surface to NEA, followed by dosing with MP. Docking complexes were identified on the surface and the imaging temperature was sufficiently low that the complexes were stable and no dynamic behaviour was observed in consecutive images. Typical high-resolution images of the most commonly observed types of complex in which NEA is coordinated to a single MP molecule, are shown in Fig. 3a–c. The complexes were classified by measuring the angle subtended between the region of the NEA molecule due to the naphthyl ring (indicated by yellow lines) and the long axis of the MP molecule adjacent to it (indicated by red lines; Supplementary Fig. 19). In rare cases, two MP molecules were found to coordinate to NEA. An example of this structure is shown in the wider-scan-area image in Fig. 3(d), which also displays well-separated, isolated NEA adsorbed as exo (highlighted in red) and endo (highlighted in blue) conformers. Statistical analyses were performed for multiple images for over 100 clearly identifiable complexes (see Supplementary Discussion for the measurement protocol and sampling error calculations). Other complexes could have been identified in more crowded regions of the surface, but were not included in the statistical analysis because of the difficulty in unequivocally distinguishing a MP molecule from an adjacent naphthyl ring. In all cases, MP was found to adsorb adjacent to the ethylamine functionality.

**Figure 4:**
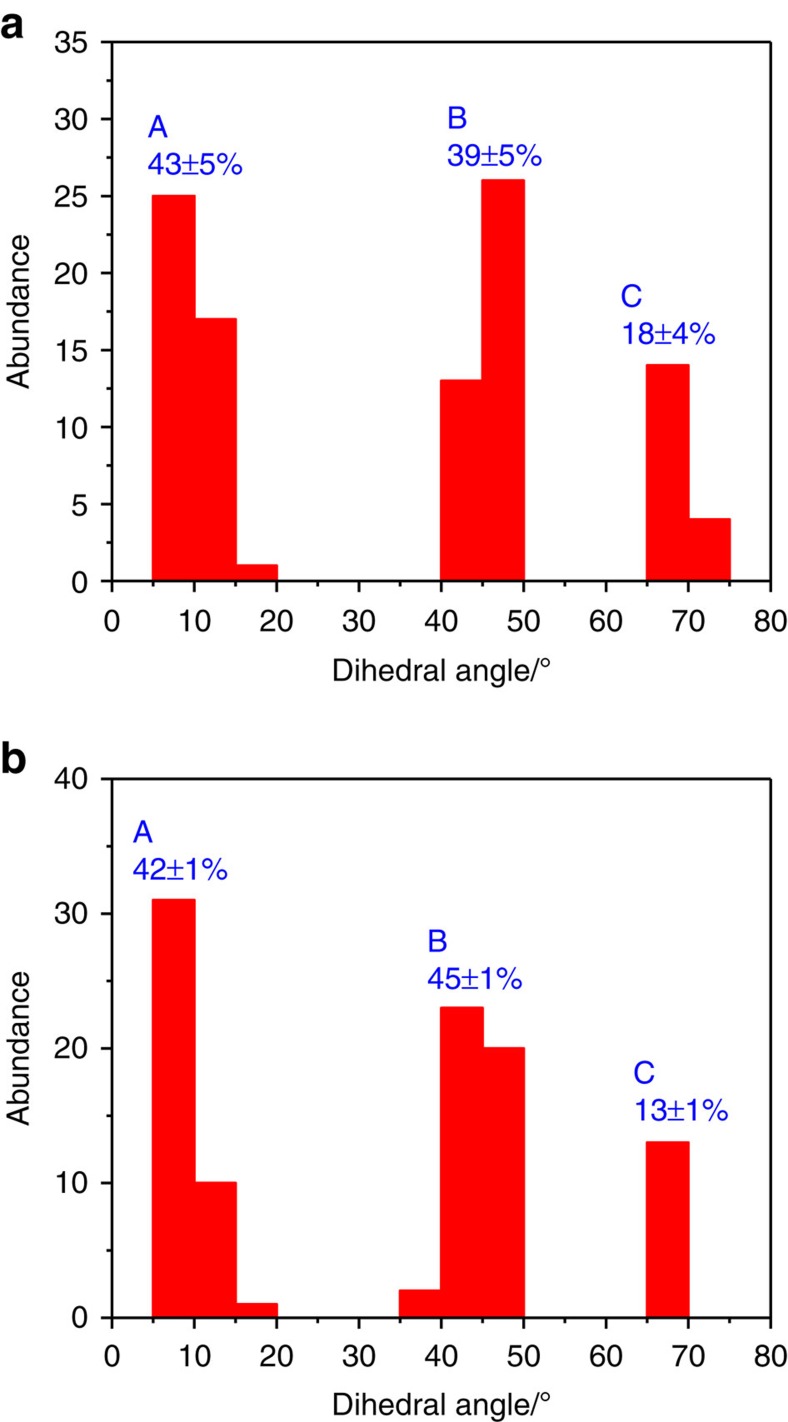
Comparison of experimental and calculated distributions of docking complexes formed between MP and NEA on Pd(111). The figure displays histograms showing the proportions of (**a**) experimentally measured and (**b**) calculated abundances versus the dihedral angles between the naphthyl ring of NEA and the long axis of MP. The results reveal that the complexes fall into three, well-defined bins defined by a range of dihedral angles; A; 10±5°, B; 45±5° and C; 70±5°.

A histogram showing the experimental distribution of the abundance of the docking complexes is displayed as a function of the measured angles in Fig. 4a. The dihedral angles fall into three well-defined bins designated **A** (10±5°), **B** (45±5°) and **C** (70±5°). The difference between the angles that define each bin is sufficiently large that the images of clearly defined complexes could be unequivocally assigned to each bin. The measured populations are summarized in Table 1 with the associated sampling errors. Approximately 3% of the docking complexes were found to include two MP molecules oriented at 10±5 and 70±5° to the NEA naphthyl ring (Fig. 3d). It was not possible to unequivocally establish the NEA isomer contained in each of the docking complexes due to the proximity of the MP bonded to it.

## Discussion

Co-adsorption of MP with NEA enhances the hydrogenation rate of MP to methyl lactate (Fig. 1), thereby reproducing the effect found for catalytic systems. Adsorbed MP also undergoes significant H-D exchange to form d_3_-MP (Fig. 2) both on clean Pd(111) and the NEA-covered surface. Such H-D exchange indicates reaction with the e*nol* tautomer on the surface. H-D exchange also occurs on Pd(111) in the absence of co-adsorbed NEA, but the proportion of MP undergoing complete exchange increases on NEA-modified Pd(111) (Fig. 2, Inset).

While the STM images identify docking complexes and provide structural information, the resolution is insufficient to distinguish the forms of NEA and MP. Accordingly, the docking of *enol* and *keto* tautomers of MP with both the endo and exo isomers of NEA was investigated using DFT calculations by constructing isolated 1:1 complexes of all four combinations with MP adsorption constrained to be proximate to the chiral ethylamine centre on NEA. MP becomes chiral due to removal of the mirror plane by adsorption and can therefore adopt Pro-*R* or Pro-*S* configurations. Calculations were therefore carried out for the interaction of both MP enantiomers with NEA. Initial calculations were performed without including van der Waals' interactions. However, since van der Waals forces have been found to provide a large contribution to the binding energies on coinage metal surfaces^50^, the energies and structures of the most stable docking complexes were then calculated by including van der Waals interactions using the method of Tkatchenko and Scheffler^51^, which leads to more stable structures and larger interaction energies in all cases. While DFT calculations of binding energies can have significant systematic errors, it is expected that the relative energies of the same systems in different configurations will be reasonably accurate. Interaction energies were calculated relative to the exo and endo conformers of NEA and the *keto* tautomer of MP alone on a 6 × 6 Pd(111) slab (Supplementary Discussion). These results provide an indication of the relative interaction strengths of various docking complexes. Interaction energies of the most stable docking complexes were then calculated by including van der Waals interactions (Supplementary Table 12). The resulting energies were then used to calculate relative equilibrium proportions of each docking complex at a sample temperature of 120 K. Since STM images were collected from multiple experiments over a period of time, the effect of temperature fluctuations of ±10 K on the equilibrium distributions was also estimated.

Finally, the STM images of the most stable structures were simulated using the Tersoff–Hamman approach^52^ (Supplementary Figs 16 and 17). In all cases, the shapes of the simulated images agree with the experimental images. The dihedral angles between MP and the naphthyl ring of NEA in the simulated images were measured in the same way as for the experiment and fell into the same ranges. The equilibrium distribution of angles in the simulated images of the calculated structures are displayed in Fig. 4b and summarized in Table 1, where the errors include the uncertainly in temperature discussed above. Including docking complexes arising from just the exo conformer of NEA yields distributions that are not in good agreement with experiment, while adding complexes with both endo and exo NEA agrees very well. This indicates that the correct structures are reproduced by the DFT calculations and thus provides atomic-scale information on diastereomeric interactions between MP and NEA.

**Figure 5:**
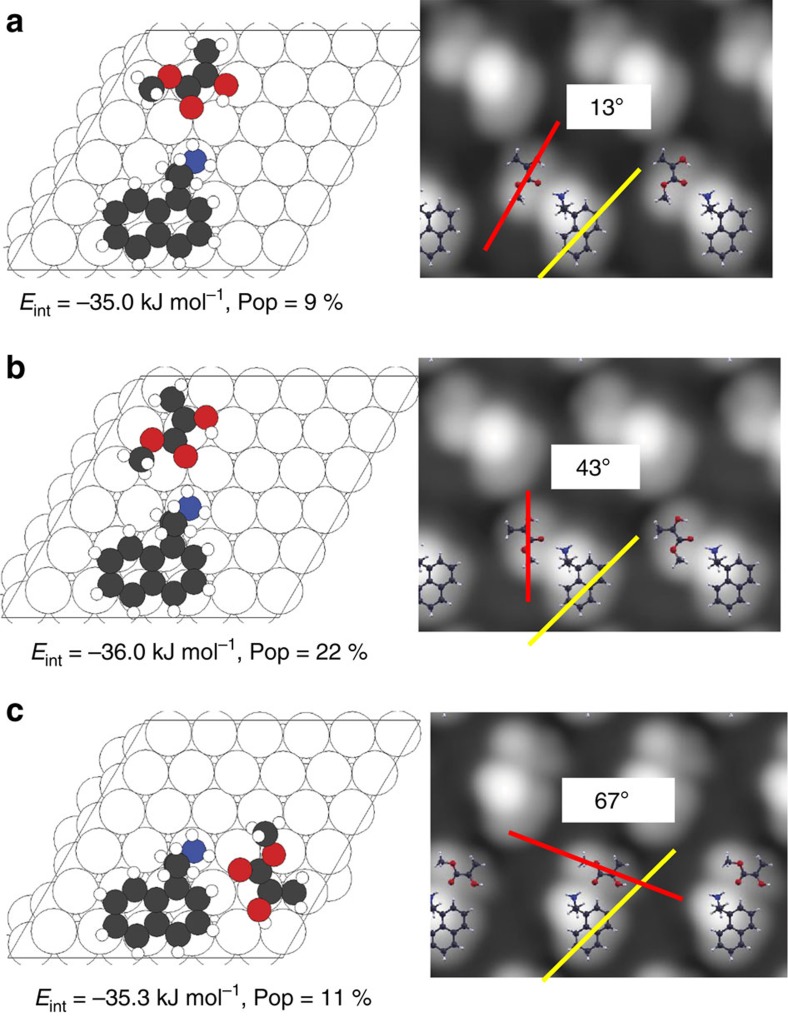
Calculated structures of the docking complexes that form between exo-NEA and MP on Pd(111). Depictions of DFT-optimized structures of the most stable docking complexes formed between MP and exo-NEA adsorbed on the dibridge[6] site, indicated as **a**–**c**. The interaction energies calculated including van der Waals' interactions are shown below each structure. The STM images simulated by the Tersoff–Hamman method are shown adjacent to each structure. The angles between the axis in the simulated images of the *enol* tautomer of MP, indicated by red lines, and the long axis of the naphthyl group of NEA, indicated by yellow lines, are also indicated. The relative population of each type of docking complex is calculated using a Boltzmann distribution and is reported below the respective structures.

**Figure 6:**
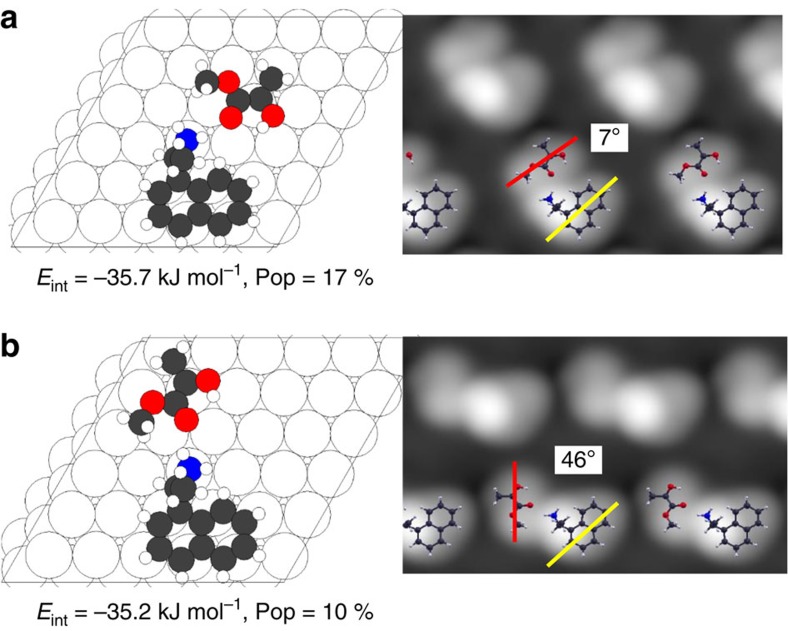
Calculated structures of the docking complexes that form between endo-NEA and MP on Pd(111). Depictions of DFT-optimized structures of the most stable docking complexes formed between MP and endo-NEA adsorbed on the dibridge[6] site, indicated as **a** and **b**. The interaction energies calculated including van der Waals' interactions are shown below each structure. The STM images simulated by the Tersoff–Hamman method are shown adjacent to each structure. The angles between the axis in the simulated images of the *enol* tautomer of MP, indicated by red lines, and the long axis of the naphthyl group of NEA, indicated by yellow lines, are also indicated. The relative population of each type of docking complex is calculated using a Boltzmann distribution and is reported below the respective structures.

The most stable docking complexes (those with greater than ∼8% abundance) are displayed for exo NEA in Fig. 5 and for endo NEA in Fig. 6, while the remainder of the docking complexes that appear less frequently, but which were nevertheless included in the calculation of the populations, are shown in Supplementary Figs 16 and 17, along with their interaction energies and calculated equilibrium proportions. Figures 5 and 6 also show the simulated STM images and indicate both the calculated interaction energies and equilibrium proportions.

The majority of the docking complexes are with the *enol* tautomer of MP (78%), with the remainder interacting with the *keto* form. Thus, the enhanced hydrogenation activity of NEA-covered Pd(111) compared with the clean surface (Fig. 1) is ascribed to the preferential docking of the *enol* form of MP where the C=C double bond undergoes more facile hydrogenation than the C=O bond^40^. The participation of the *enol* tautomer has been proposed previously for MP hydrogenation on cinchonidine- and cinchonine-modified, supported palladium catalysts^53^. This conclusion is in accord with the similarity in peak temperature for MP hydrogenation on NEA-covered Pd(111) and for ethylene hydrogenation on clean Pd(111) (Supplementary Fig. 1). As noted above, MP can be classified into whether reacts to form *R*- or *S*-methyl lactate and, assuming only the *eno*l form hydrogenates, predicts an ee of *R*-methyl lactate in the presence of *R*-NEA of ∼92%. The calculated proportion of the docking complexes that form with the exo isomer of NEA is ∼54%, with the remainder docking with the endo form. This ratio is close to that found for NEA adsorbed alone on Pd(111), where the 57±3% adsorbs as the exo conformer^31^.

All the most stable complexes (Figs 5 and 6) bind to the *enol* MP tautomer in a *cis* configuration^38^, with the C=C double bond located at Pd–Pd bridge sites. The methylene hydrogens are distorted from a planar geometry as found for ethylene on Pd(111)^54^. Docking is dominated by C=O····H_2_N hydrogen-bonding interactions, with the majority of the most stable complexes arising from interactions between the CH_3_–C–C=O group of MP and one of the two amine hydrogens of NEA at an optimal hydrogen-bonding distance of ∼0.22 nm (Supplementary Fig. 20). Thus, the most stable complexes are predominantly those which allow bridge-bonding of the vinyl group while simultaneously optimizing O····HN hydrogen bonding, although dipole-dipole interactions between NEA and MP play a role (Supplementary Discussion).

The proposed docking complexes obey the three-point bonding rule found for biological systems^55^,^56^. Here, three-point bonding occurs between the two vinyl group carbons that preferentially bind to atop sites on Pd(111) and by a C=O····H_2_N hydrogen-bonding interaction between the *enol* MP tautomer and an amine group on the chiral centre. Three-point bonding has also been observed between *R*-glycidol adsorbed on *S*,*S*-bitartrate-modified Pd(111)^57^. Here, the epoxide oxygen on glycidol adsorbs on a palladium atop site which interacts by a simultaneous hydrogen donation from the CH_2_–OH group to the carboxylate, and a hydrogen acceptor interaction between the CH_2_–OH group and a hydroxyl group of the bitartrate. Thus, two of the three bonds in the NEA–MP interaction are to the surface and one with the modifier, while in the glycidol-bitartrate system, glycidol binds to the surface through the epoxide oxygen to an atop palladium site, with two stereo-directing interactions with the modifier. Chemisorption energies to surfaces are typically stronger than hydrogen-bonding interactions, so that surface bonding dictates the most stable locations of both the chiral modifier and chiral species or prochiral reactants. The most favourable one-to-one interactions of a chiral modifier with a chiral probe (as in the case of glycidol) or a prochiral reactant (in the case of MP) are predominantly influenced by the way in which their binding to the surface controls the stereochemistry of the weaker hydrogen-bonding interactions between them. Such a molecular-level understanding of chiral and diastereomeric interactions with chiral modifiers will lead to the rational design of heterogeneous chiral catalysts.

The interaction energies of the most stable docking complexes on Pd(111) vary between ∼35 and 37 kJ mol^−1^ (Figs 5 and 6) while the fluorinated *keto* analogue of MP, methyl 3,3,3-trifluoropyruvate (MTFP), where *enol* formation is suppressed^36^, interacts more strongly (with interaction energies between ∼52 and ∼62 kJ mol^−1^) with NEA on Pt(111)^45^. This emphasizes the importance of either the substrate or fluorination in controlling complexation and, in the case of MTFP, docking complexes are observed at ∼300 K on platinum, while lower temperatures (∼120 K) are needed to observe the complexes on palladium.

The stabilization of the *enol* tautomer results in the formation of a C=C double bond that hydrogenates more easily than does a carbonyl group, resulting in enhanced MP hydrogenation on NEA-modified Pd(111) (Fig. 1). Ethylene hydrogenates by a sequential addition of atomic hydrogen, by the so-called Horiuti–Polanyi mechanism^58^. H/D exchange occurs through the half-hydrogenated ethyl intermediate, which repeatedly undergoes β-hydride elimination reactions to incorporate deuterium. Ethane is formed by hydrogenating the ethyl intermediate. A similar mechanism for hydrogenation of the vinyl group of the *enol* tautomer of MP implies that the enhancement in H/D exchange in MP can be ascribed to its stabilization by NEA, and the enhancement in methyl lactate formation indicates that the half-hydrogenated MP is also stabilized.

Stable docking complexes are found to form between the *enol* tautomer of MP with both the exo and endo rotamers of NEA, predicted both by first-principles DFT calculations that include van der Waals interactions and is also confirmed by comparing experimental STM images with Tersoff–Hamman simulations of the calculated structures^52^. The formation of the complexes is primarily dictated by the surface ensemble requirements of binding the carbon–carbon double bond to the surface while simultaneously optimizing C=O····H_2_N hydrogen-bonding interactions that predominantly stabilizes the *enol* tautomer in pro-*R* configurations on an *R*-NEA-modified surface. The stabilization of the *enol* tautomer results in enhanced hydrogenation of MP to methyl lactate due to the presence of a more easily hydrogenated C=C double bond. MP hydrogenation, therefore, essentially proceeds in two steps; an initial stabilization of the *enol* tautomer of MP to form a C–OH single bond and a C=C double bond, followed by facile C=C bond hydrogenation to yield MP. The combination of chiral-NEA driven diastereomeric docking with a tautomeric preference causes an enhancement of hydrogenation activity that will lead to increased enantiomeric excesses in the catalytic reaction for chirally modified surfaces, as found experimentally. This model provides a rationale for the catalytic observations in which enhanced hydrogenation rates are found for chirally modified surfaces. However, care must be taken in directly transferring observations made for model systems in ultrahigh vacuum to supported catalysts operating in more severe solution environments, where, for example, different modifier adsorption geometries may occur^59^.

## Methods

### Sample cleaning and preparation procedure

The Pd(111) substrate was cleaned using a standard procedure consisting of cycles of argon ion sputtering and annealing in 3 × 10^−8^ Torr of oxygen at 1,000 K. (*R*)-(+)-α-(1-naphthyl)ethylamine (Acros Organics, 99% purity) was dosed from a home-built Knudsen source described elsewhere^30^. Because of the high vapour pressure of NEA, the Knudsen source was cooled to ∼200 K for at least 60 min before dosing. MP (Alfa Aesar, 98% purity) was purified by several freeze-pump-thaw cycles. After dosing NEA, the sample was cooled to ∼120 K and held at that temperature for all imaging. MP was dosed onto the Pd(111) surface through a variable leak valve while maintaining a sample temperature of ∼120 K.

### STM imaging procedure

STM images were acquired at a sample temperature of ∼120 K using an electrochemically etched tip made from recrystallized tungsten wire. This was conditioned by a controlled interaction with a clean Au(111) single-crystal surface, likely resulting in a gold-terminated tip^60^. Experiments were performed using a STM (RHK UHV350 dual AFM/STM) housed in an ultrahigh vacuum chamber operating at a base pressure below 2 × 10^−10^ Torr following bakeout, as described elsewhere^41^.

### TPD experiments

TPD data were collected in another chamber operating at a base pressure of 8 × 10^−11^ Torr, using a linear heating rate of 2.5 K s^−1^, where desorbing species were monitored using a Dycor quadrupole mass spectrometer placed close to and in-line-of-sight of the sample.

### LEED experiments

LEED data were collected in a doubly μ-metal-shielded vacuum chamber to minimize extraneous magnetic fields, and pumped by means of a liquid-nitrogen trapped diffusion pump, which operated at a base pressure of 2 × 10^−10^ Torr following bakeout. I/V curves were collected with a four-grid retarding-field analyzer and the diffraction patterns were recorded as a function of beam voltage using a digital camera^54^,^61^,^62^,^63^.

### Computational methods

DFT calculations were performed with the projector augmented wave method^64^,^65^ as implemented in the Vienna Ab-initio simulation package (VASP) (refs 66, 67, 68). The exchange-correlation potential was described using the generalized gradient approximation of Perdew, Burke and Ernzerhof^69^, and hydrogen-bonding interactions are reasonably well reproduced (within∼4 kJ mol^−1^) using this functional, although the accuracy deteriorates as the hydrogen bonds deviate from linear^70^. A cutoff of 400 eV was used for the planewave basis set, and the wavefunctions and electron density were converged to within 1 × 10^−5^ eV. The first Brillouin zone was sampled with a 3 × 3 × 1 Γ-centred k-point mesh. Geometric relaxations were considered to be converged when the force was <0.02 eV Å^−1^ on all unrestricted atoms. Van der Waals corrections were included by using the method of Tkatchenko and Scheffler^51^. STM topography simulations were performed with the Tersoff-Hamman^52^ approach as implemented in bSKAN 3.7, which has been found to reproduce the shapes of experimental STM images for MP on Pd(111)^38^ as well as those obtained using the more rigorous Bardeen approach^71^.

### Data availability

The data that support the findings of this study are available from the authors upon request.

## Additional information

**How to cite this article:** Mahapatra, M. *et al*. Enhanced hydrogenation activity and diastereomeric interactions of methyl pyruvate co-adsorbed with *R*-1-(1-Naphthyl)ethylamine on Pd(111). *Nat. Commun.* 7:12380 doi: 10.1038/ncomms12380 (2016).

## Supplementary Material

Supplementary Information Supplementary Figures 1-20, Supplementary Tables 1-14, Supplementary Discussion and Supplementary References.

## Figures and Tables

**Table 1:** Comparison between measured and calculated proportions of docking complexes.

**Dihedral angle range/**°	**Measured proportion/%**	**Calculated proportion, exo NEA only/%**	**Calculated proportion, exo and endo NEA/%**
10±5	43±5	28	42±1
45±5	39±5	48	45±1
70±5	18±4	24	13±1

Measured proportions of NEA–MP docking complexes that have dihedral angles in the STM images between the naphthyl group of NEA and the long axis of the MP of 10±5, 45±5 and 70±5°, compared with the results of the image analysis of the structures of the docking complexes calculated using DFT for only the exo form of NEA, and then including both the endo and exo forms. The measured proportions include the sampling error and the calculated proportions take into account possible variations in the sample temperature.
