# Supplementary material for: Enhanced hydrogenation activity and diastereomeric interactions of methyl pyruvate co-adsorbed with R-1-(1-naphthyl)ethylamine on Pd(111)
Source: Nat Commun. 2016 Aug 4;7:12380. doi: 10.1038/ncomms12380 (PMC4976220; doi:10.1038/ncomms12380)
Supplement: Supplementary Information — Supplementary Figures 1-20, Supplementary Tables 1-14, Supplementary Discussion and Supplementary References. [file ncomms12380-s1.pdf]

Supplementary Figures

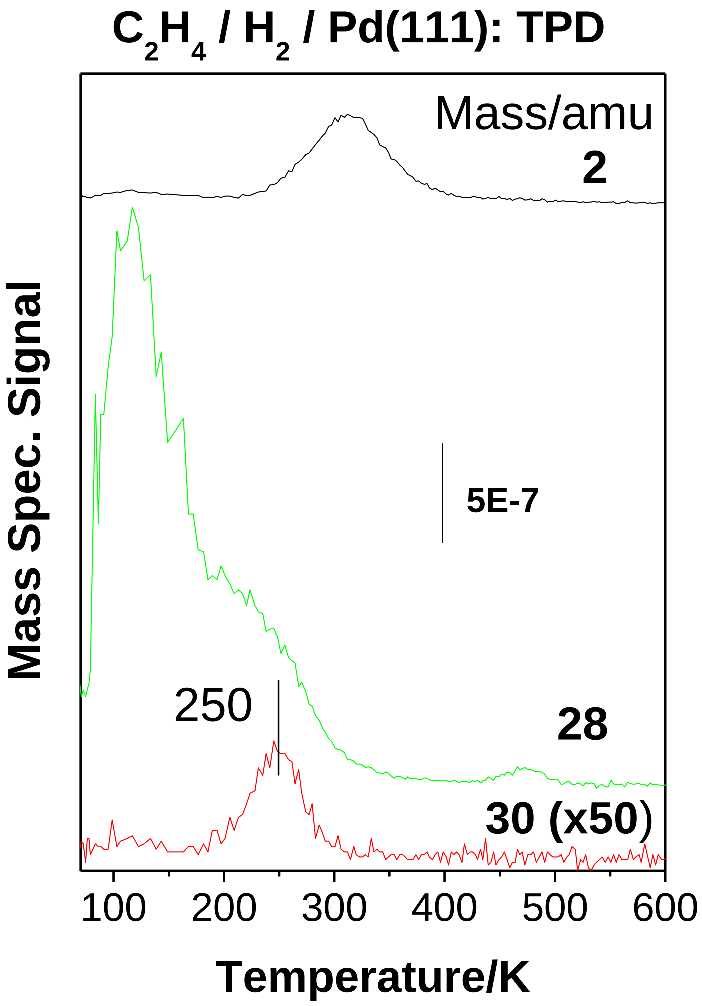

Supplementary Figure 1: Temperature-programmed desorption of 2 L of C_2H_4 adsorbed onto a hydrogen pre-covered Pd(111) surface (2 L of H_2) at 80 K collected using a heating rate of 3 K/s, monitoring 2 (H_2), 28 (C_2H_4) and 30 (C_2H_6) amu.

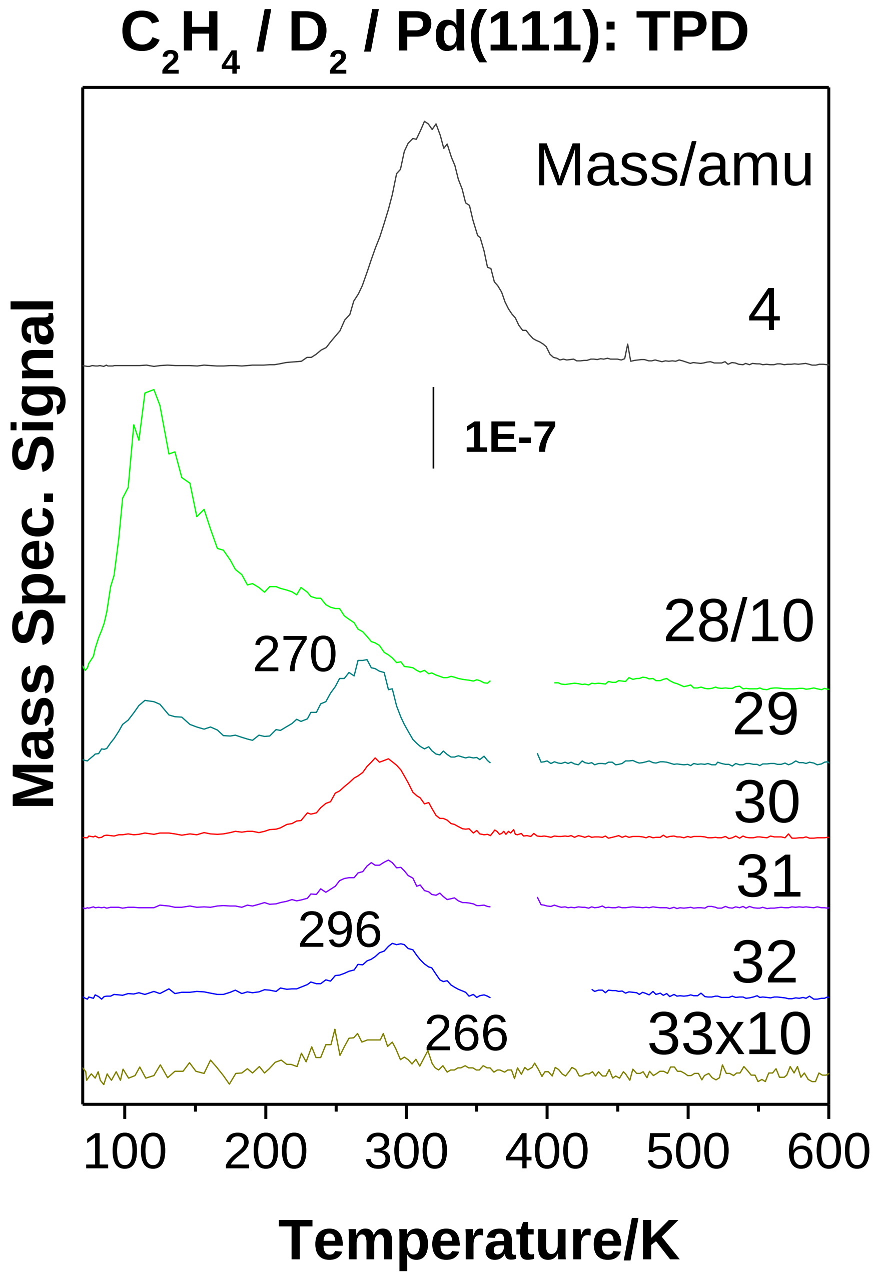

Supplementary Figure 2: Temperature-programmed desorption of 2 L of C_2H_4 adsorbed onto a deuterium pre-covered Pd(111) surface (2 L of D_2) at 80 K collected using a heating rate of 3 K/s, monitoring various masses, indicated adjacent to the corresponding spectrum.

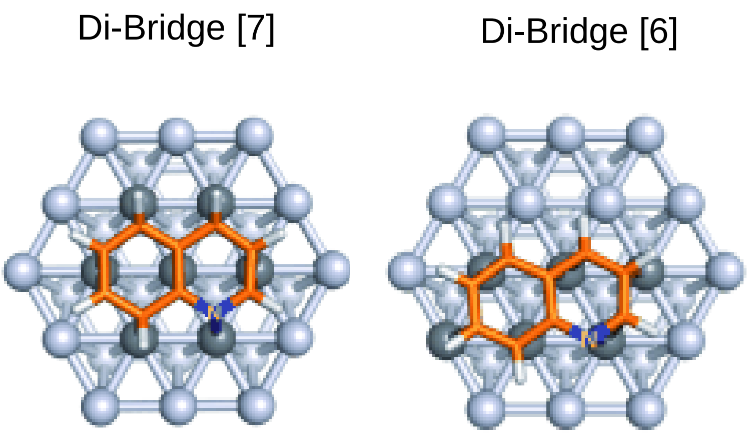

Supplementary Figure 3: Illustration of the dibridge[6] and dibridge[7] naphthalene adsorption sites on Pd(111).

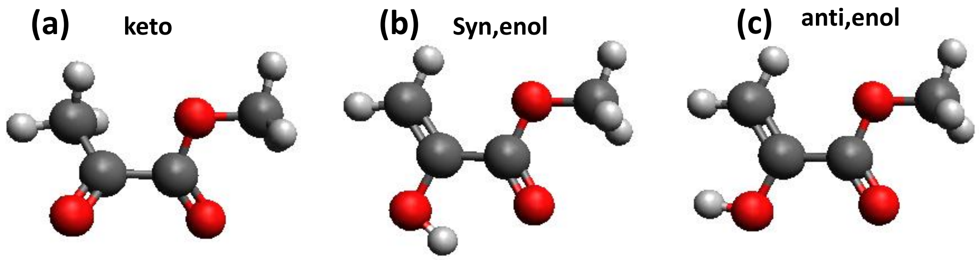

Supplementary Figure 4: Structures of various forms of methyl pyruvate: (a) *keto*, (b) syn, *enol* (c) anti, *enol*.

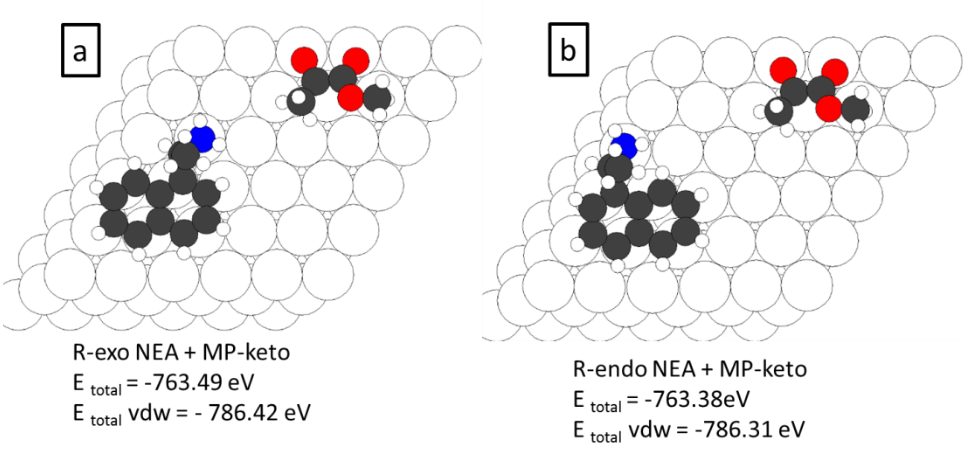

Supplementary Figure 5: Reference structures of (a) *R-exo* NEA + MP-*keto* (b) *R-endo* NEA + MP-*keto.*

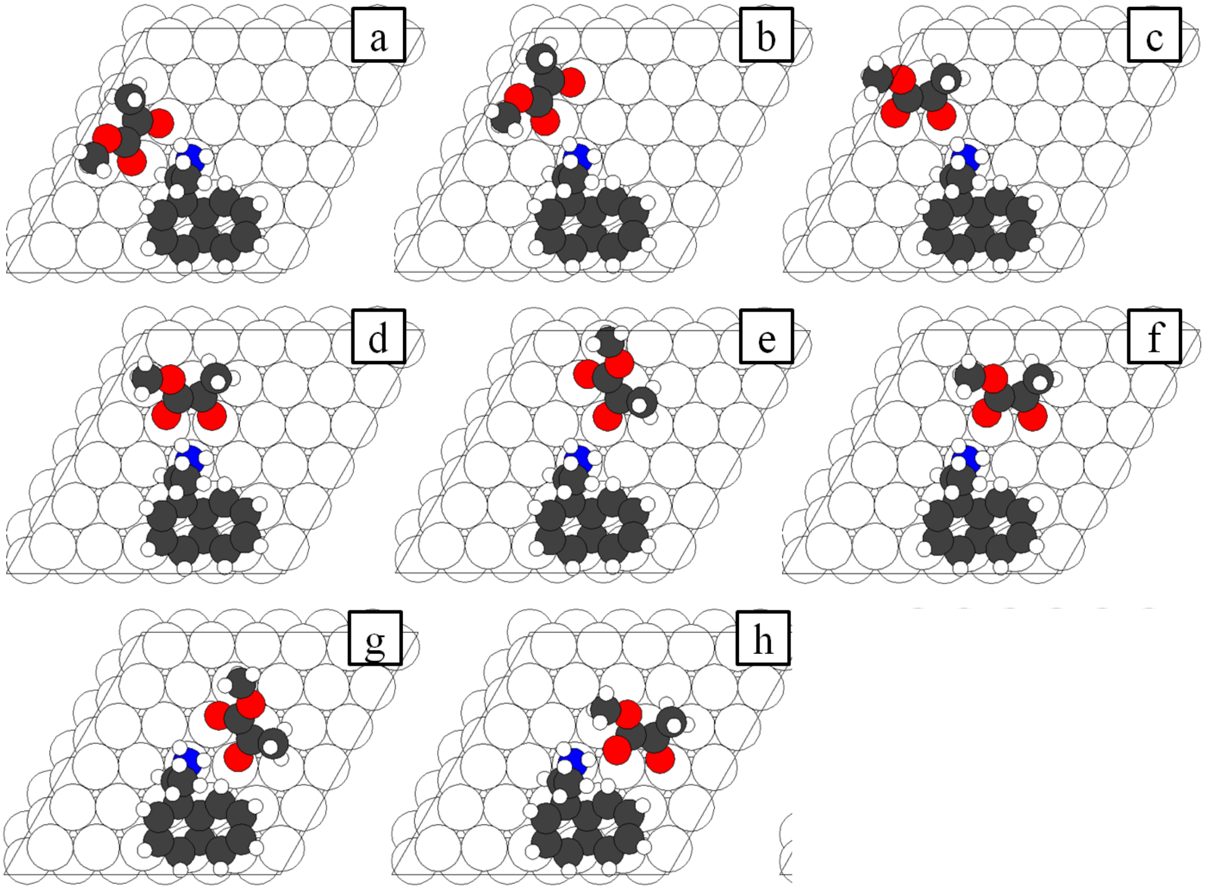

Supplementary Figure 6: Converged geometries for combinations of *R-endo* NEA and pro-R *keto* methyl pyruvate on Pd(111) from DFT calculations. They are identified as: (a) Geo01, (b) Geo02, (c) Geo03, (d) Geo04, (e) Geo05, (f) Geo06, (g) Geo07 and (h) Geo08.

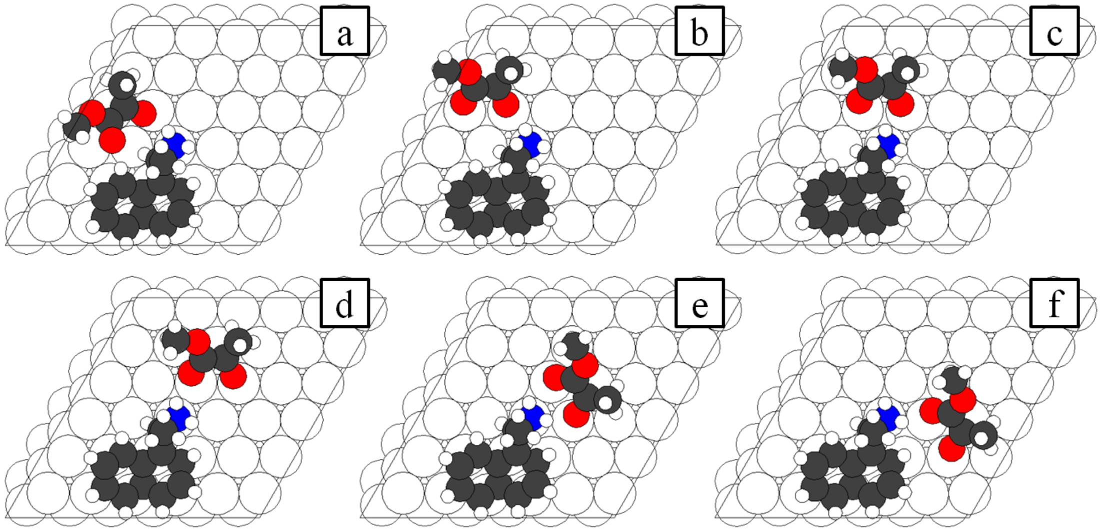

Supplementary Figure 7: Converged geometries for combinations of *R-exo* NEA and pro-R *keto* methyl pyruvate on Pd(111) from DFT calculations. They are identified as: (a) Geo01, (b) Geo02, (c) Geo03, (d) Geo04, (e) Geo05 and (h) Geo06.

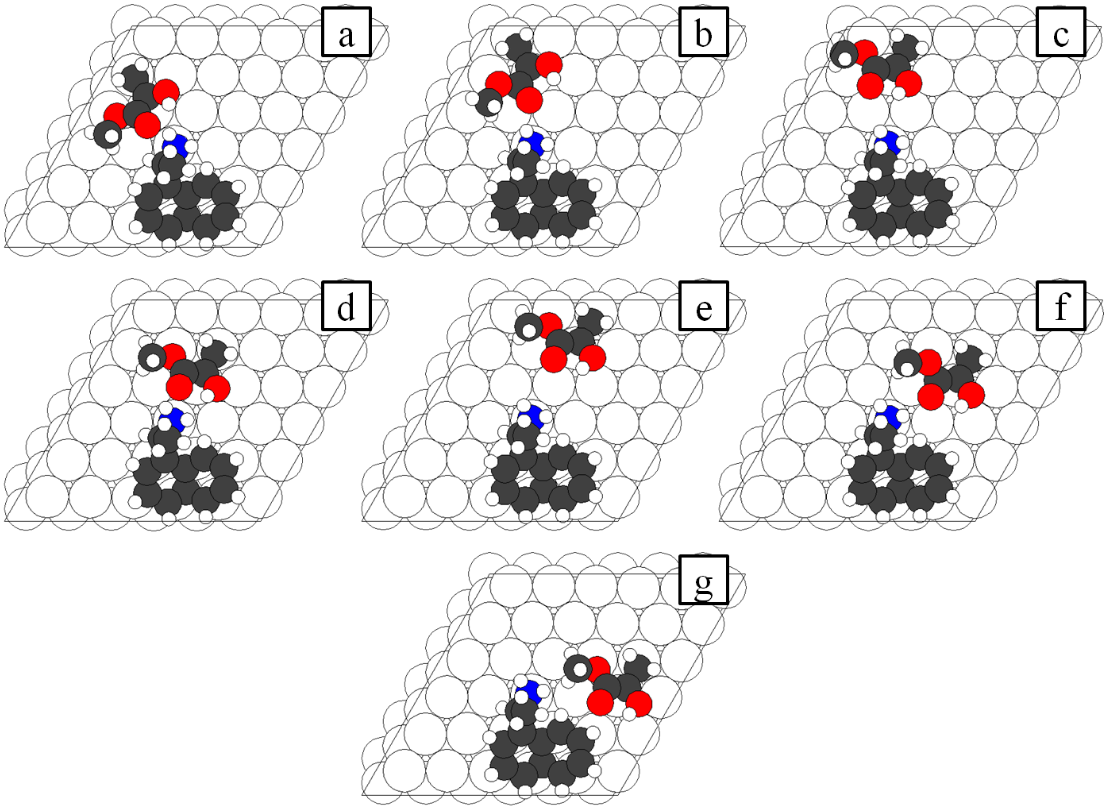

Supplementary Figure 8: Converged geometries for combinations of *R-endo* NEA and pro-R syn, *enol* methyl pyruvate on Pd (111) from DFT calculations. They are identified as: (a) Geo01, (b) Geo02, (c) Geo03, (d) Geo04, (e) Geo05, (f) Geo06 and (g) Geo07.

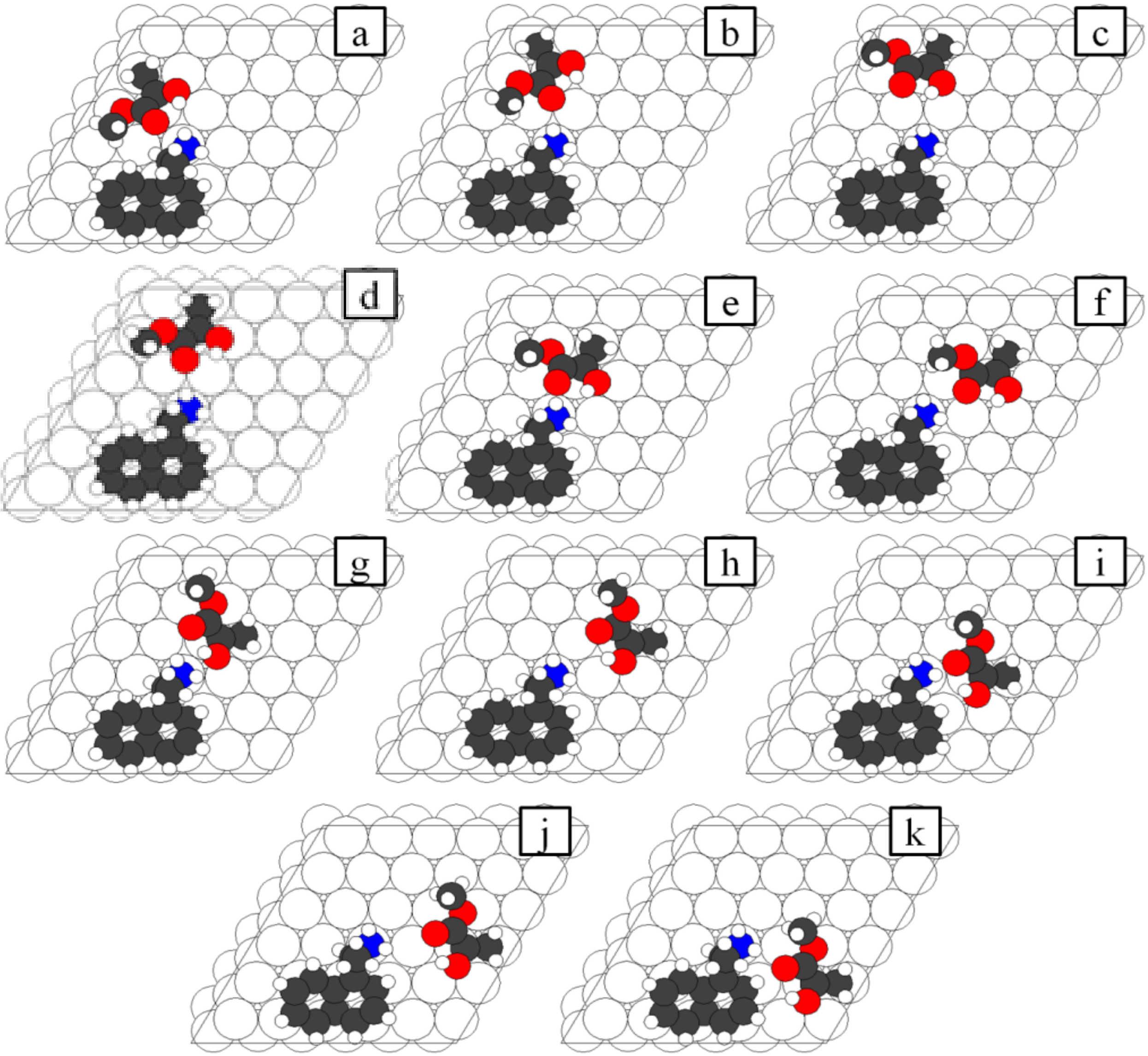

Supplementary Figure 9: Converged geometries for combinations of *R-exo* NEA and pro-R syn, *enol* methyl pyruvate on Pd(111) from DFT calculations. They are identified as: (a) Geo01, (b) Geo02, (c) Geo03, (d) Geo04, (e) Geo05, (f) Geo06, (g) Geo07, (h) Geo08, (i) Geo09, (j) Geo10 and (k) Geo11.

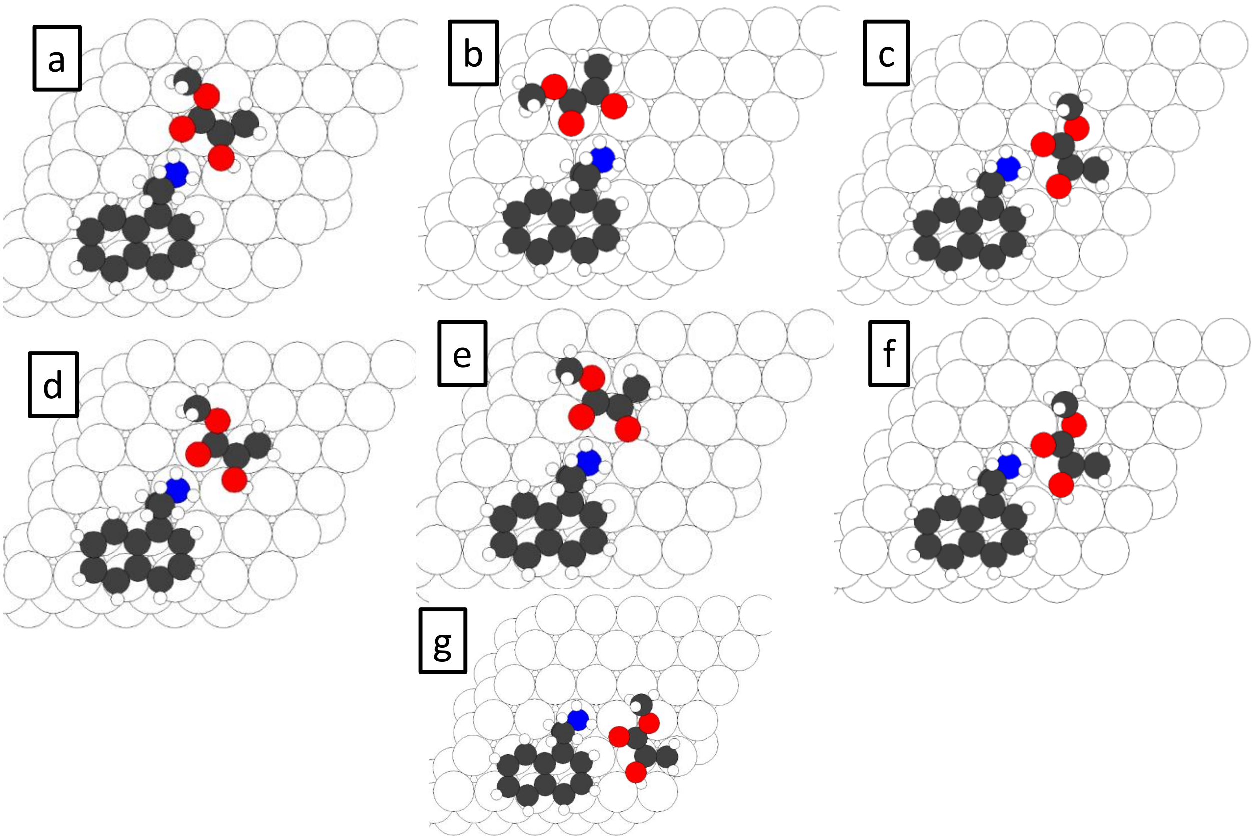

Supplementary Figure 10: Converged geometries for combinations of *R-exo* NEA and pro-R anti-*enol* methyl pyruvate on Pd(111) from DFT calculations. They are identified as: (a) Geo01, (b) Geo02, (c) Geo03, (d) Geo04, (e) Geo05, (f) Geo06 and (g) Geo07.

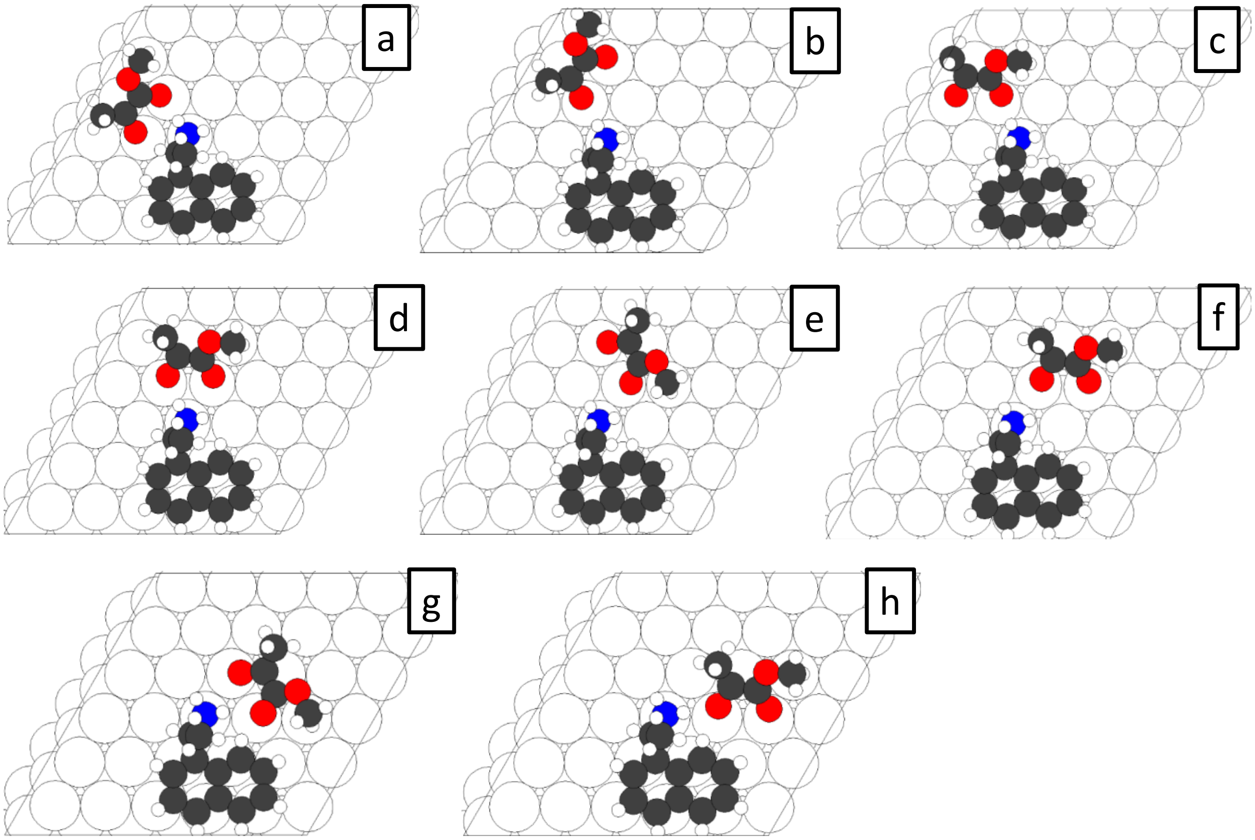

Supplementary Figure 11: Converged geometries for combinations of *R-endo* NEA and pro-S *keto* methyl pyruvate on Pd(111) from DFT calculations. They are identified as: (a) Geo01, (b) Geo02, (c) Geo03, (d) Geo04, (e) Geo05, (f) Geo06, (g) Geo07 and (h) Geo08.

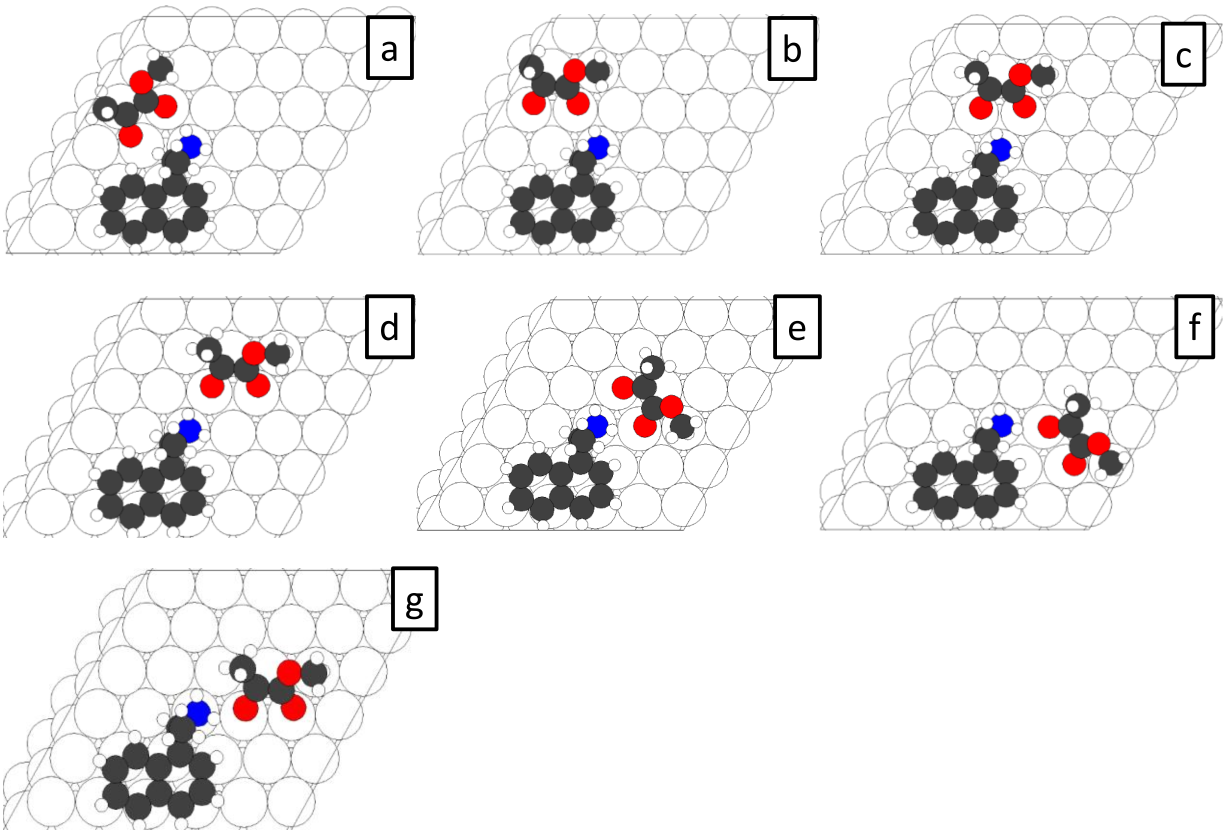

Supplementary Figure 12: Converged geometries for combinations of *R-exo* NEA and pro-S *keto* methyl pyruvate on Pd(111) from DFT calculations. They are identified as: (a) Geo01, (b) Geo02, (c) Geo03, (d) Geo04, (e) Geo05, (f) Geo06 and (g) Geo07.

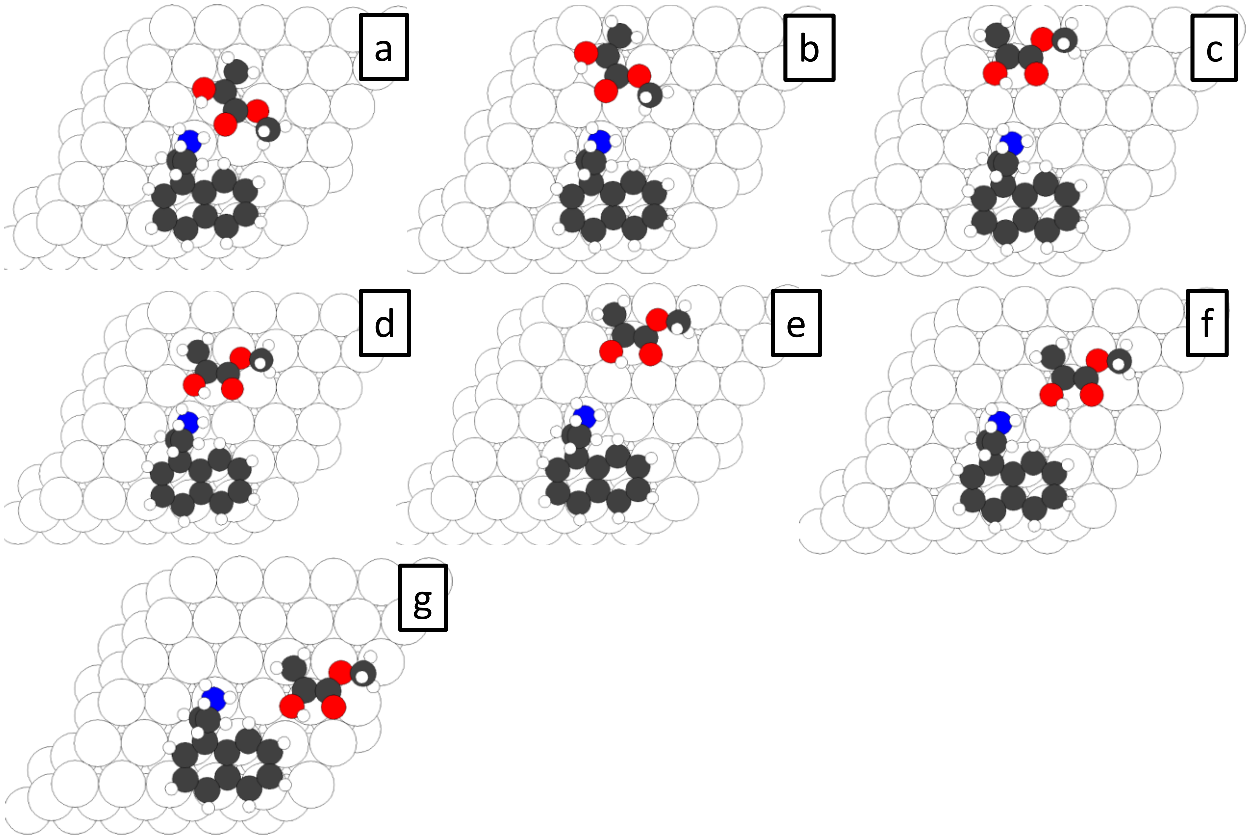

Supplementary Figure 13: Converged geometries for combinations of *R*-*endo* NEA and pro-S syn *enol* methyl pyruvate on Pd(111) from DFT calculations. They are identified as: (a) Geo01, (b) Geo02, (c) Geo03, (d) Geo04, (e) Geo05, (f) Geo06 and (g) Geo07.

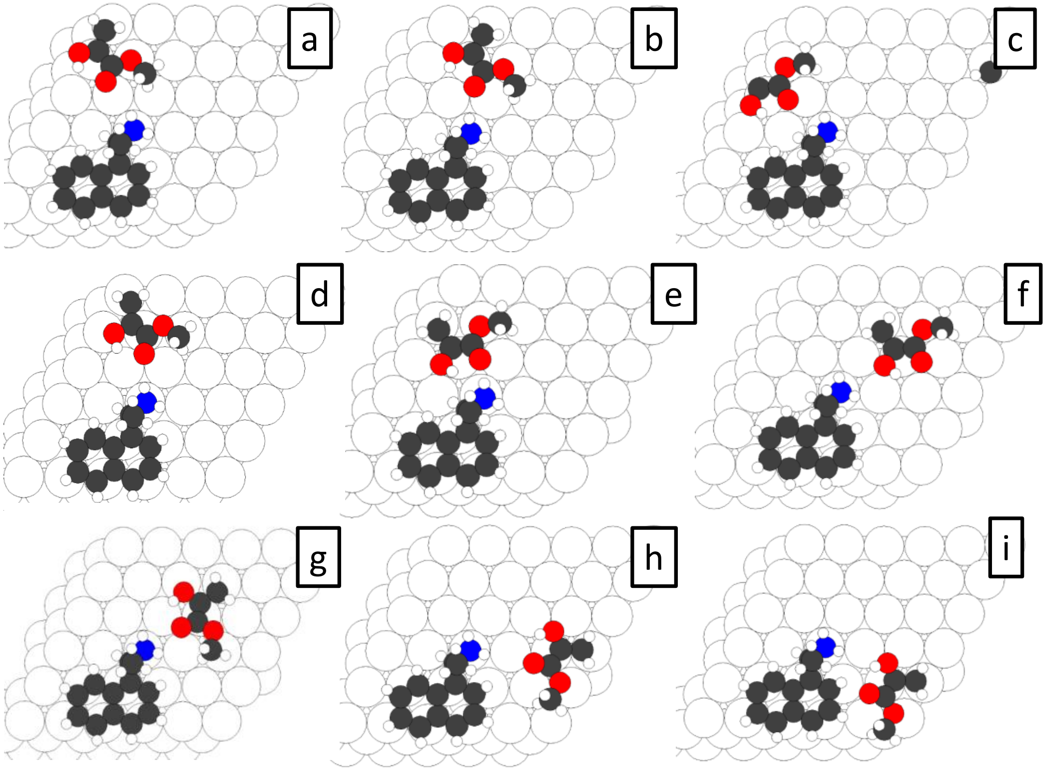

Supplementary Figure 14: Converged geometries for combinations of *R-exo* NEA and pro-S syn *enol* methyl pyruvate on Pd(111) from DFT calculations. They are identified as: (a) Geo01, (b) Geo02, (c) Geo03, (d) Geo04, (e) Geo05, (f) Geo06, (g) Geo07, (h) Geo08 and (i) Geo09.

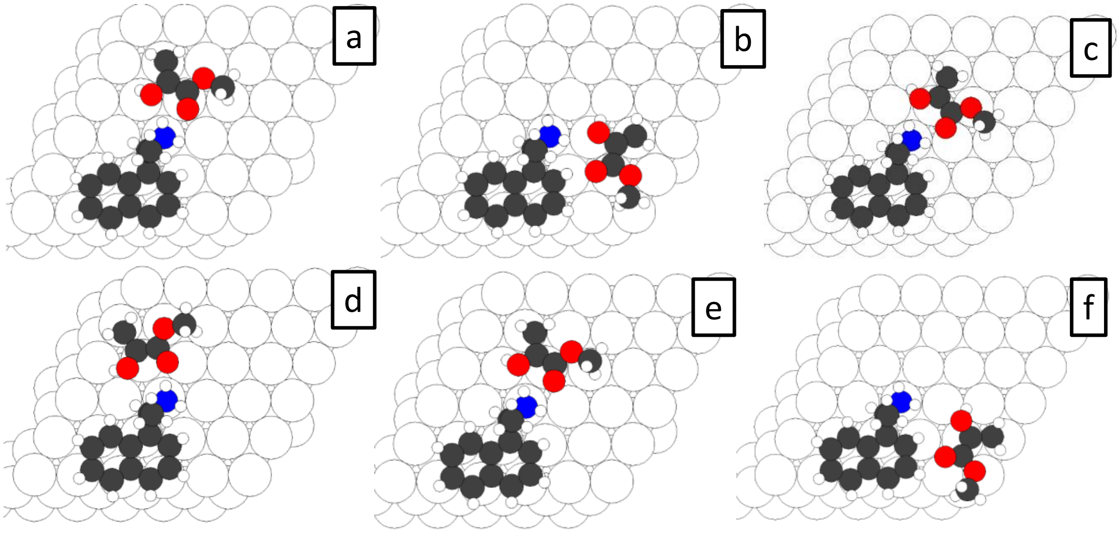

Supplementary Figure 15: Converged geometries for combinations of *R-exo* NEA and pro-S anti *enol* methyl pyruvate on Pd(111) from DFT calculations. They are identified as: (a) Geo01, (b) Geo02, (c) Geo03, (d) Geo04, (e) Geo05 and (f) Geo06.

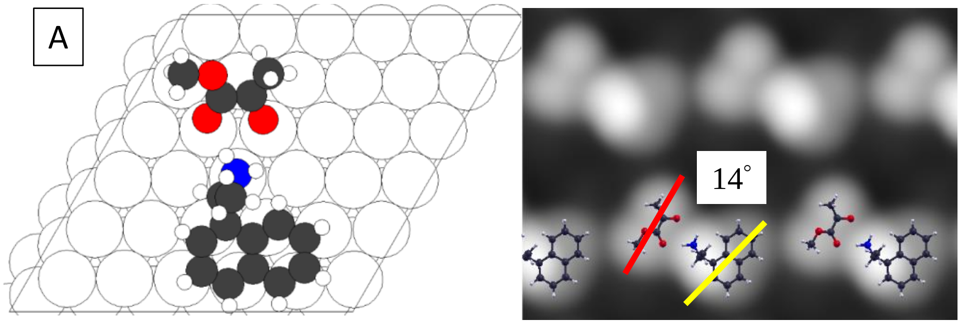

E $_{int}$ = -31.3kJ/mol, Pop = 0.2 %

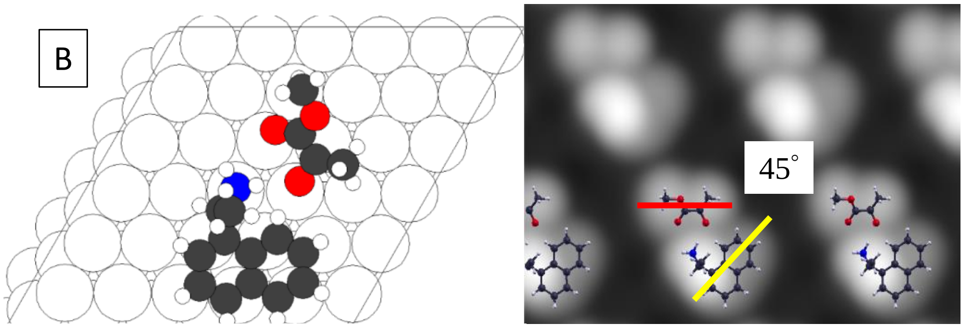

E $_{int}$ = -35.1kJ/mol, Pop = 9.1 %

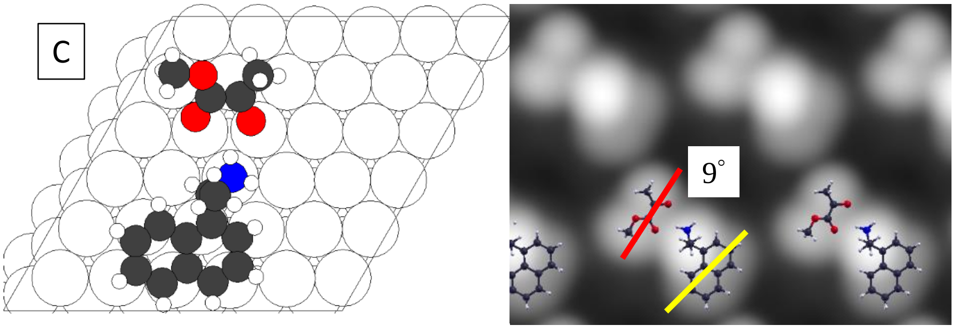

E $_{int}$ = -32.5kJ/mol, Pop = 0.7 %

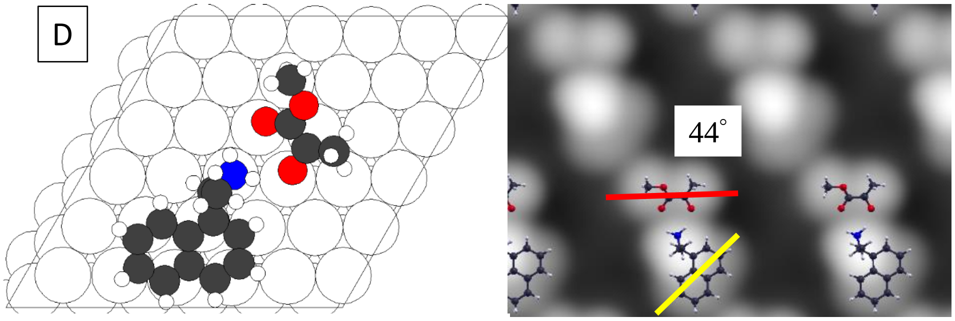

E $_{int}$ = -32.9 kJ/mol, Pop = 1.1 %

E

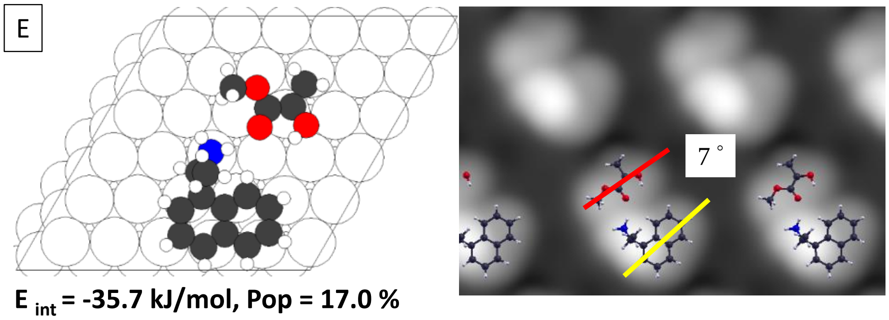

E $_{int}$ = -35.7 kJ/mol, Pop = 17.0 %

F

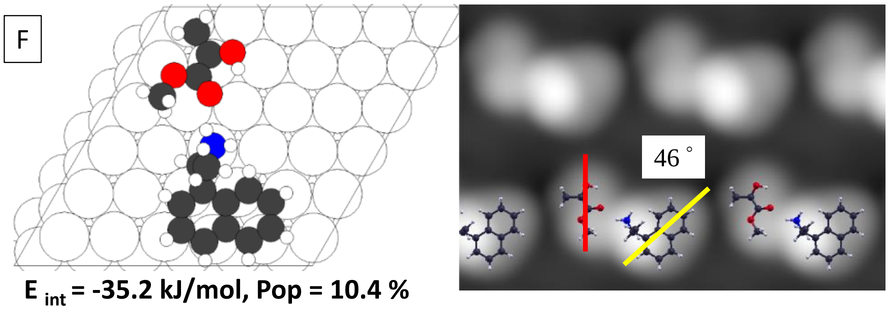

E $_{int}$ = -35.2 kJ/mol, Pop = 10.4 %

G

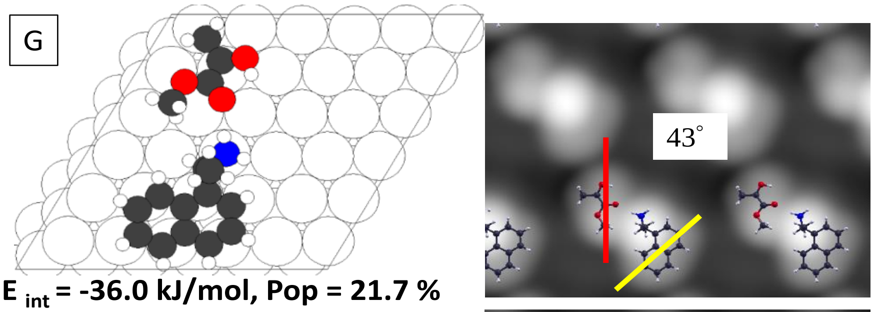

E $_{int}$ = -36.0 kJ/mol, Pop = 21.7 %

H

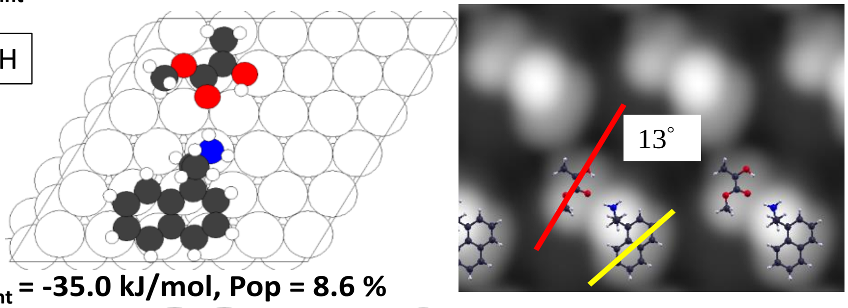

E $_{int}$ = -35.0 kJ/mol, Pop = 8.6 %

I

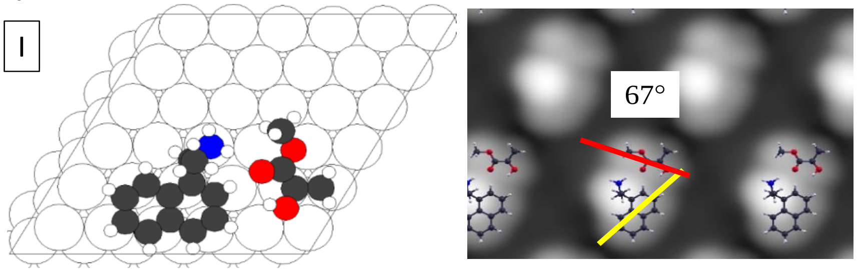

E $_{int}$ = -33.6 kJ/mol, Pop = 2.0 %

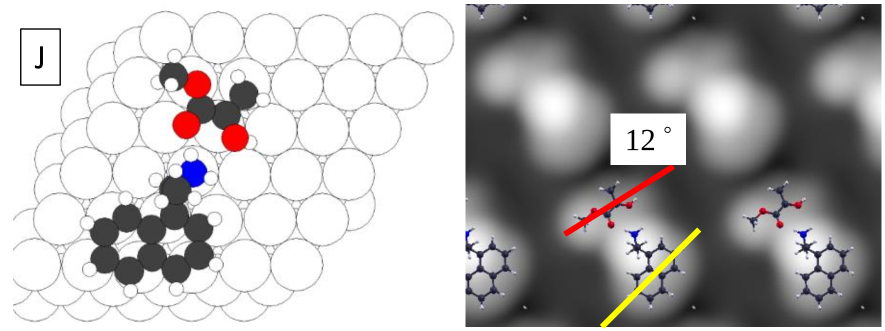

E $_{int}$ = -33.3 kJ/mol, Pop = 1.4 %

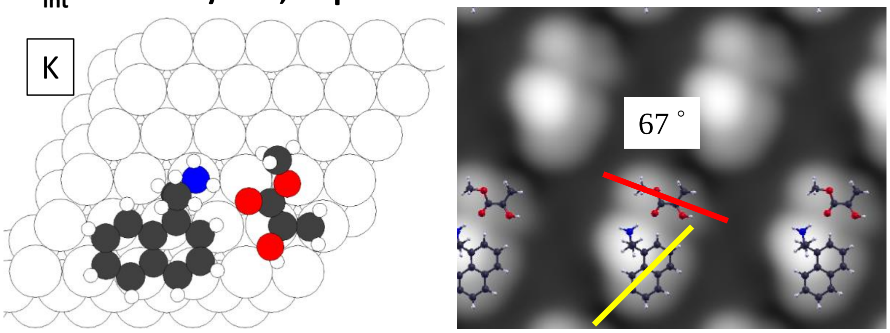

E $_{int}$ = -35.3 kJ/mol, Pop = 10.8 %

Supplementary Figure 16: Structures of the most stable docking complexes from the pro-*R* MP and *R*-NEA. The docking complexes are formed between *endo* NEA and *keto* MP in A and B, *exo* NEA and *keto* MP in C and D, *endo* NEA and syn, *enol* MP in E and F, *exo* NEA and syn, *enol* MP in G, H and I, *exo* NEA and anti, *enol* MP in J and K. The interaction energies calculated including van der Waals' interactions are shown below each structure, as well as the calculated equilibrium proportions. The STM images simulated by the Tersoff-Hamman method are shown adjacent to each structure. The angles between the axis in the simulated images of the MP, indicated by a red line, and the long axis of the naphthyl group of *R*-NEA, indicated by a yellow line, are also indicated.

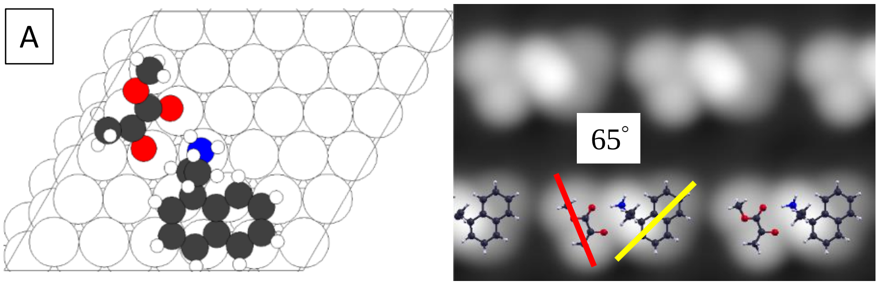

E $_{int}$ = -32.1 kJ/mol, Pop = 0.4 %

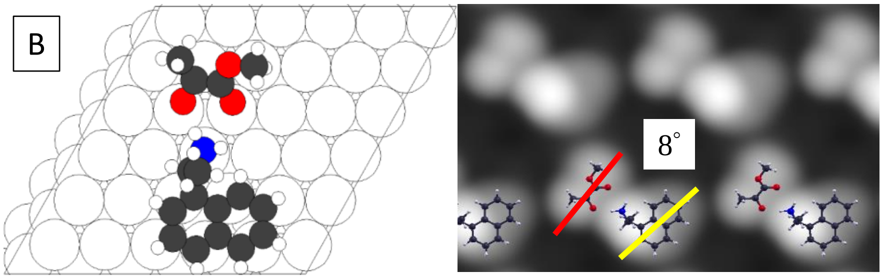

E $_{int}$ = -34.1 kJ/mol, Pop = 3.3 %

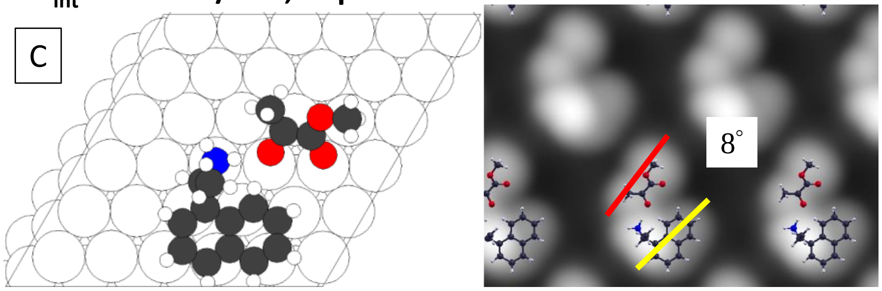

E $_{int}$ = -34.7 kJ/mol, Pop = 5.9 %

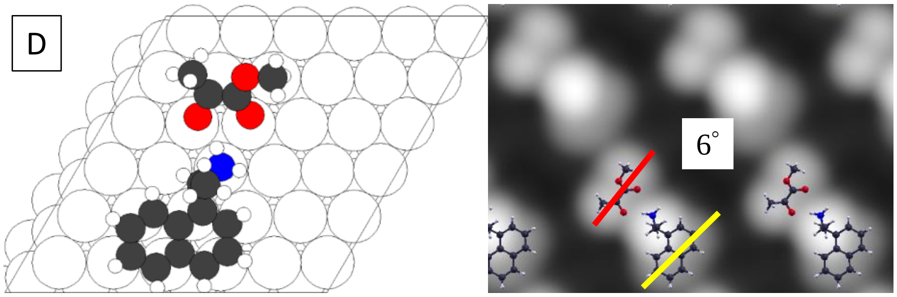

E $_{int}$ = -31.3 kJ/mol, Pop = 0.2 %

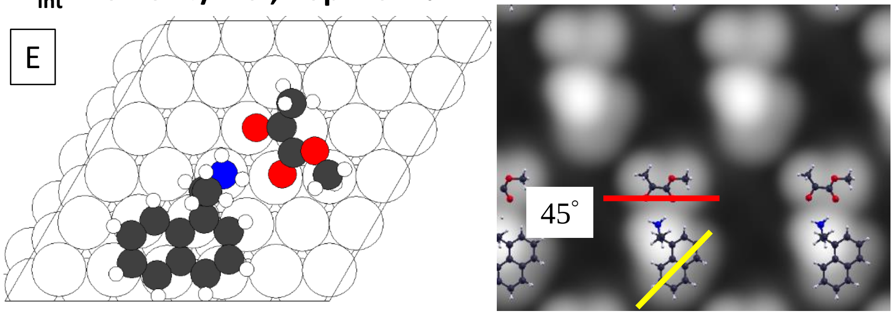

E $_{int}$ = -32.6 kJ/mol, Pop = 0.7 %

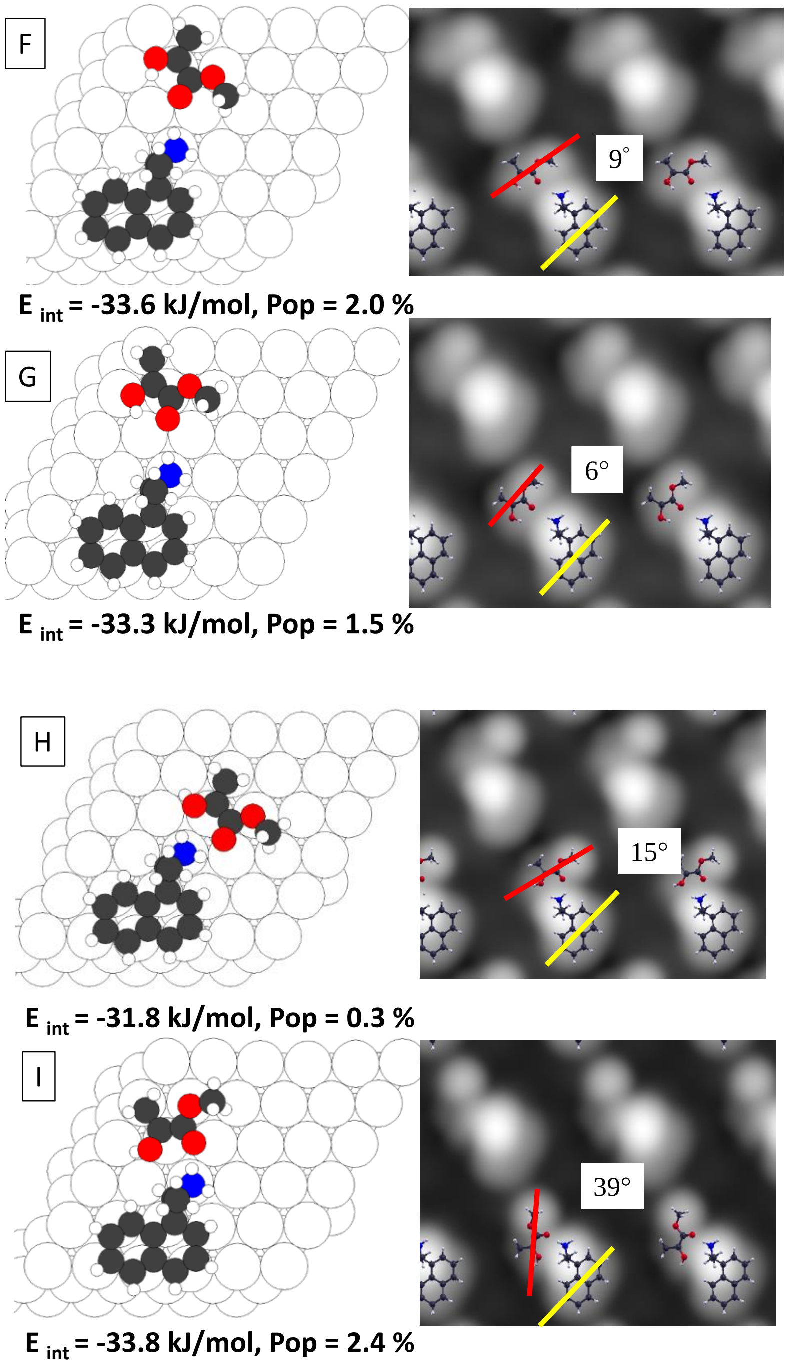

Supplementary Figure 17: Structures of the most stable docking complexes from the pro-*S* MP and *R*-NEA. The docking complexes are formed between *endo* NEA and *keto* MP in A, B and C, *exo* NEA and *keto* MP in D and E, *exo* NEA and syn, *enol* MP in F and G, *exo* NEA and anti, *enol* MP in H and I. The interaction energies calculated including van der Waals' interactions are shown below each structure, as well as the calculated equilibrium proportions. The STM images simulated by the Tersoff-Hamman method are shown adjacent to each structure. The angles between the axis in the simulated images of the MP, indicated by a red line, and the long axis of the naphthyl group of *R*-NEA, indicated by a yellow line, are also indicated.

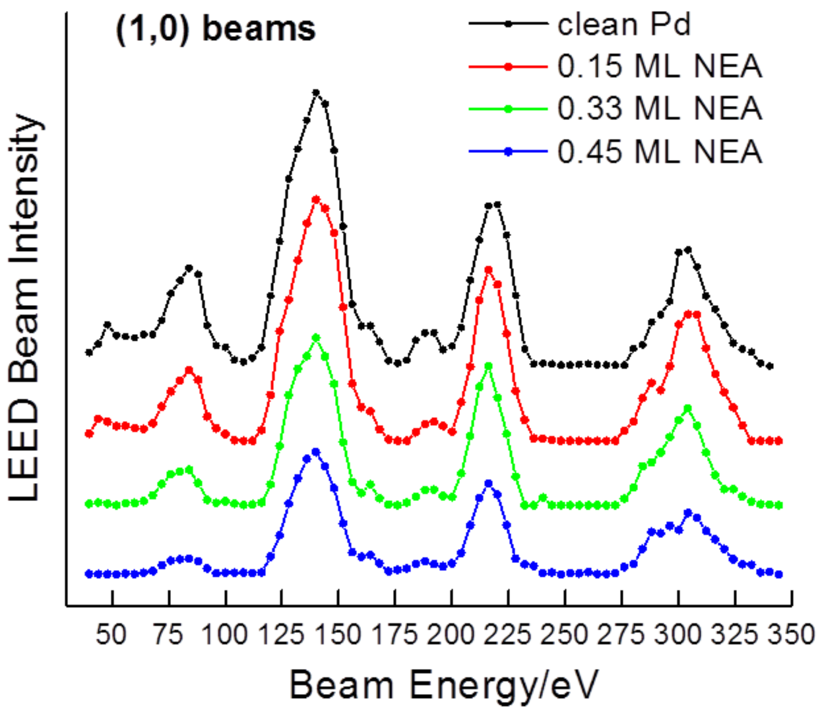

Supplementary Figure 18: LEED I/V curve measurements of clean Pd(111) and Pd(111) surface dosed with various coverages of *S*-NEA. The I/V curves shown in the Figure represent the (1,0) beams.

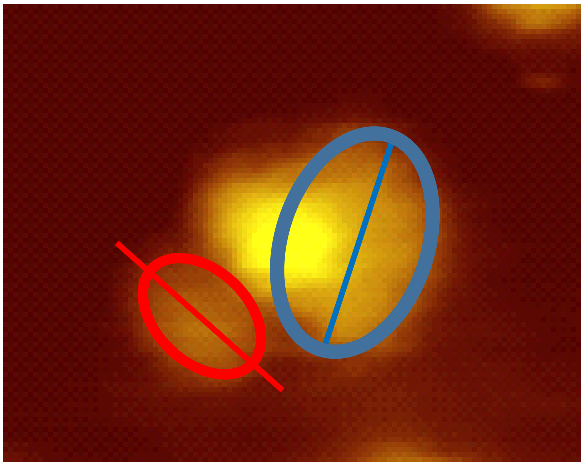

Supplementary Figure 19: Illustration of the criteria used to define the relative orientations of the naphthyl ring of NEA and the long axis of the adjacent MP.

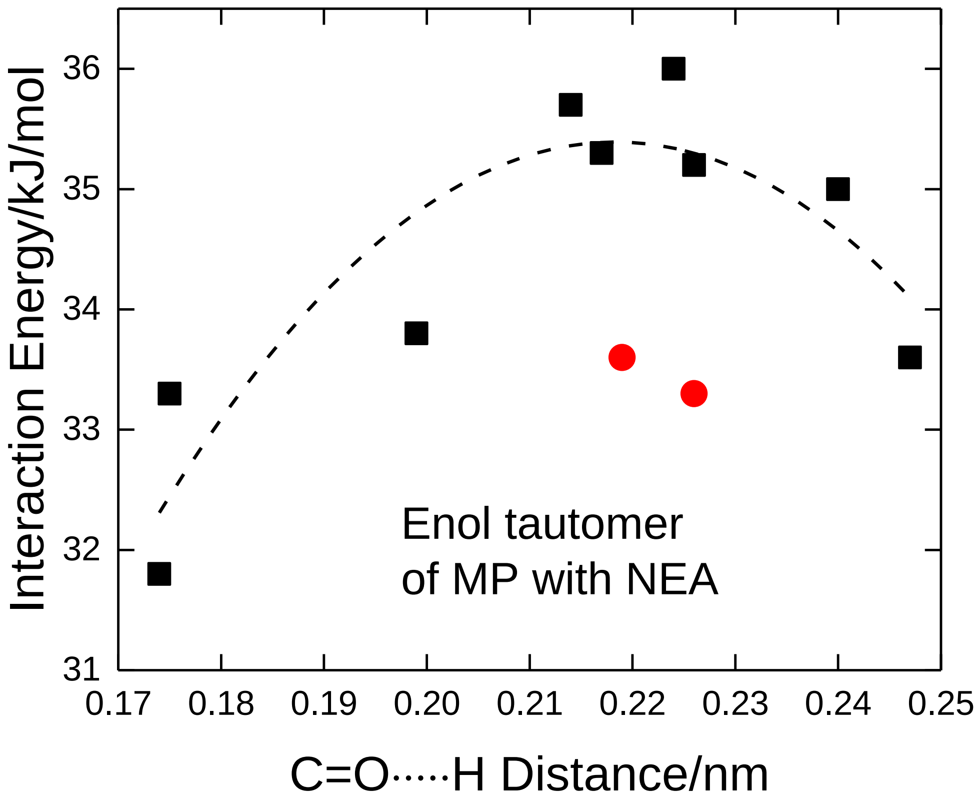

Supplementary Figure 20: Plot of the interaction energy of the most stable docking complexes between mostly Pro-*R* MP and *R*-NEA as a function of C=O···H_2N distance (■). The red points (●) highlight docking complexes that deviate significantly from the trend line. The line is shown as a guide to the eye.

Supplementary Tables

Supplementary Table 1: Proportion of deuterated ethylene isotopomers from reaction of C_2H_4 on deuterium-covered Pd(111) taken from Supplementary Figure 2.

Deuterated ethylene	Proportion/%
C_2H_3D	35
$C_2H_2D_2$	16
C_2HD_3	24
C_2D_4	25

Supplementary Table 2: Interaction energies of *R-endo* NEA and pro-R *keto* methyl pyruvate on Pd(111) from DFT calculations for the geometries shown in Supplementary Figure 6.

**System**	**E_{int} (kJ/mol)**	**E_{int} (kJ/mol) Vdw**
***R*-endo NEA + pro-R MP keto**		
Geo01	-22.8	-29.4
Geo02	-13.9	
Geo03	-18.8	
Geo04	-26.1	-31.3
Geo05	-11.8	
Geo06	-9.8	
Geo07	-25.9	-35.1
Geo08	-21.0	-25.7

Supplementary Table 3: Interaction energies of *R-exo* NEA and pro-R *keto* methyl pyruvate on Pd(111) from DFT calculations for the geometries shown in Supplementary Figure 7.

**System**	**E_{int} (kJ/mol)**	**E_{int} (kJ/mol) Vdw**
***R*-exo NEA + pro-R MP keto**		
Geo01	-13.3	
Geo02	-19.4	-21.7
Geo03	-24.8	-32.5
Geo04	-11.5	
Geo05	-27.7	-32.9
Geo06	-18.5	-20.8

Supplementary Table 4: Interaction energies of *R-endo* NEA and pro-R syn, *enol* methyl pyruvate on Pd(111) from DFT calculations for the geometries shown in Supplementary Figure 8

**System**	**E_{int} (kJ/mol)**	**E_{int} (kJ/mol) Vdw**
***R*-endo NEA + pro-R MP enol**		
Geo01	3.7	
Geo02	-26.1	-35.2
Geo03	-12.6	
Geo04	-0.7	
Geo05	-15.7	
Geo06	-27.0	-35.7
Geo07	-5.4	

Supplementary Table 5: Interaction energies of *R-exo* NEA and pro-R syn, *enol* methyl pyruvate on Pd(111) from DFT calculations for the geometries shown in Supplementary Figure 9

System	E_{int} (kJ/mol)	E_{int} (kJ/mol) Vdw
***R*-exo NEA + pro-R MP-enol**		
Geo01	9.0	
Geo02	-28.0	-36.0
Geo03	-11.6	
Geo04	-27.9	-35.0
Geo05	-10.9	
Geo06	-21.7	-27.3
Geo07	8.6	
Geo08	-11.7	
Geo09	-4.7	
Geo10	-16.7	-17.6
Geo11	-22.5	-33.6

Supplementary Table 6: Interaction energies of *R-exo* NEA and pro-R anti-*enol* methyl pyruvate on Pd(111) from DFT calculations for the geometries shown in Supplementary Figure 10

System	E_{int} (kJ/mol)	E_{int} (kJ/mol) Vdw
***R*-exo NEA + pro-R anti, MP- enol**		
Geo01	-9.8	
Geo02	-5.1	
Geo03	-12.5	
Geo04	-4.3	
Geo5	-23.8	-33.3
Geo6	-12.9	-26.0
Geo7	-24.7	-35.3

Supplementary Table 7: Interaction energies of *R-endo* NEA and pro-S *keto* methyl pyruvate on Pd(111) from DFT calculations for the geometries shown in Supplementary Figure 11.

**System**	**E_{int} (kJ/mol)**	**E_{int} (kJ/mol) Vdw**
***R*-endo NEA + pro-S MP keto**		
Geo01	-22.5	-32.0
Geo02	-20.4	
Geo03	-14.7	
Geo04	-29.6	-34.1
Geo05	-10.1	
Geo06	-14.3	
Geo07	-20.1	-23.8
Geo08	-26.8	-34.7

Supplementary Table 8: Interaction energies of *R-exo* NEA and pro-S *keto* methyl pyruvate on Pd(111) from DFT calculations for the geometries shown in Supplementary Figure 12.

**System**	**E_{int} (kJ/mol)**	**E_{int} (kJ/mol) Vdw**
***R*-exo NEA + pro-S MP keto**		
Geo01	-12.6	
Geo02	-17.9	-21.7
Geo03	-27.5	-31.3
Geo04	-15.8	
Geo05	-28.1	-32.5
Geo06	-22.3	-24.8
Geo07	-21.4	-24.1

Supplementary Table 9: Interaction energies of *R-endo* NEA and pro-S syn-*enol* methyl pyruvate on Pd(111) from DFT calculations for the geometries shown in Supplementary Figure 13.

System Pro-S	E_{int} (kJ/mol)	E_{int} (kJ/mol) Vdw
***R*-endo NEA + pro-S MP syn enol**		
Geo01	-9.0	
Geo02	-22.2	-29.7
Geo03	-9.9	
Geo04	9.6	
Geo05	-8.7	
Geo06	-17.1	-22.5
Geo07	-3.9	

Supplementary Table 10: Interaction energies of *R-exo* NEA and pro-S syn-*enol* methyl pyruvate on Pd(111) from DFT calculations for the geometries shown in Supplementary Figure 14.

System	E_{int} (kJ/mol)	E_{int} (kJ/mol) Vdw
***R*-exo NEA+ pro-S syn, enol MP**		
Geo01	-8.5	
Geo02	-25.7	-33.6
Geo03	-19.4	-30.2
Geo04	-29.5	-33.3
Geo05	-11.7	
Geo06	-15.5	-18.9
Geo07	-23.2	-27.9
Geo08	-20.0	-22.0
Geo09	-13.3	-19.8

Supplementary Table 11: Interaction energies of *R-exo* NEA and anti-*enol* methyl pyruvate on Pd(111) from DFT calculations for the geometries shown in Supplementary Figure 15.

System	E_{int} (kJ/mol)	E_{int}(kJ/mol)Vdw
***R*-exo NEA + pro-S anti MP-enol**		
Geo01	0.4	
Geo02	0.9	
Geo03	-20.8	-31.8
Geo04	-21.4	-33.8
Geo05	-8.5	
Geo06	-14.3	-22.5

Supplementary Table 12: Interaction energies of the most stable docking complexes with van der Waals interactions included. The figures where the structures and simulated STM images are depicted in Supplementary Figures 16 and 17 are also indicated

NEA and Pro-R MP	E_{int} (kJ/mol)	NEA and Pro-S MP	E_{int} (kJ/mol)
	***R*-endo NEA + MP keto**		
Geo04, 16A	- 31.3	Geo01, 17A	- 32.1
Geo07, 16B	-35.1	Geo04, 17B	-34.1
		Geo08, 17C	-34.7
	***R*-exo NEA + MP keto**		
Geo03, 16C	-32.5	Geo03, 17D	-31.3
Geo05, 16D	-32.9	Geo05, 17E	-32.5
	***R*-endo NEA + MP enol, syn**		
Geo02, 16E	-35.2	-	-
Geo06, 16F	-35.7	-	-
	***R*-exo NEA + MP enol, syn**		
Geo02, 16G	-36.0	Geo02, 17F	-33.6
Geo04, 16H	-35.0	Geo04, 17G	-33.3
Geo11, 16I	-33.6		
	***R*-exo NEA + MP enol, anti**		
Geo05, 16J	-33.3	Geo03, 17H	-31.8
Geo07, 16K	-35.3	Geo04, 17I	-33.8

Supplementary Table 13: Pendry R factor values obtained from the LEED I/V analysis for clean Pd(111), NEA adsorbed on dibridge [6] and dibridge [7] sites.

.

System	Pendry R factor (Rp)
Pd(111)	0.12
Exo-NEA on dibridge[7] site	0.23
Exo-NEA on dibridge[6] site	0.14
(exo+endo) NEA on dibridge [7] site	0.23
(exo+endo) NEA on dibridge [6] site	0.15

Supplementary Table 14: The effect of dipole-dipole interactions between the Pro-S and Pro-R forms of MP and NEA. The values for ΔE(calc) are the dipole-dipole interactions calculated from Eqn. 2 and ΔE(DFT) are the energy differences between the Pro-*R* and Pro-*S* MP configurations from DFT calculations.

	Interaction Energy/kJ/mol				
Structure	OCH_3/NH_2	OH/NH_2	Total	ΔE(calc)/kJ/mol	ΔE (DFT)/kJ/mol
Fig. 17F, Pro-*S* MP	1.2	0.4	1.6	2.5	2.4
Fig. 16G, Pro-*R* MP	-1.9	1.0	-0.9		
Fig. 17G, Pro-*S* MP	1.2	0.7	1.9	1.3	1.7
Fig. 16H, Pro-*R* MP	-1.3	1.9	0.6		

Supplementary Discussion

Temperature programmed desorption of ethylene on hydrogen and duterium covered Pd(111)

The TPD profiles as shown in supplemental Figures 1 and 2 indicate that ethylene has undergone substantial hydrogen-deuterium exchange. The extent of deuterated ethane formation, as indicated by the 33 amu profile (d_3-ethane) is very small. The proportion of H-D exchange is indicated in Supplementary Table 1 using standard mass spectrometer fragmentation patterns.[1]

Density Functional Theory (DFT) Calculations of NEA/Methyl Pyruvate Docking Complexes

Previous DFT calculations of methyl pyruvate on Pd(111) have shown that it is present both in the *keto*[2, 3] and flat-lying *enol* forms.[3] Previous work suggested that NEA adsorbs with the bicyclic ring on the dibridge[7] adsorption site on Pd(111).[2] Rotation of the ethylamine group gives rise to two conformers defined as *endo* and *exo*,[2] where the *exo* conformer was found to be very slightly more stable than the *endo* conformer on the Pd(111) dibridge[7] site, by ~2 kJ/mol without van der Waals interactions, increasing to ~3 kJ/mol when they are included. However, the dibridge[6] NEA adsorption site was found to be the most stable on Pt(111) [4] with the *exo* conformer being ~13 kJ/mol more stable than the *endo* conformer. The most-stable NEA structure was therefore recalculated on the dibridge[6] site on Pd(111) and found to be slightly more stable than on the dibridge[7] site. In particular, the *exo* conformer was more stable by ~20 kJ/mol and the *endo* by 11 kJ/mol on the dibridge[6] site than the dibridge[7] site. It was also found that the *exo* conformer was calculated to be ~11 kJ/mol more stable than the *endo* conformer on the dibridge[6] site. STM images of NEA on Pd(111) reveal that the *exo* conformer occurs only slightly more often than the *endo* conformer (with a ratio of 6:4) [5] and with a ratio of 7:3 on Pt(111).[4]

Based on these results, DFT calculations were performed for combinations of both the pro-*R* and pro-*S* forms of *keto*, syn and anti *enol* forms of methyl pyruvate (shown in Supplementary Figure 4) with the two forms of NEA adsorbed on dibridge[6] sites, initially without including van der Waals interactions. For each combination of NEA and methyl pyruvate, calculations were performed with the methyl pyruvate molecule located at a range of sites around the ethylamine group of the NEA molecule. The carbonyl and alcohol oxygen atoms of the methyl pyruvate species were oriented towards the amine group of the NEA molecule.

Supplementary Figure 5 shows the reference structures of (a) *exo*-NEA with *keto* MP and (b) *endo*-NEA with the *keto* MP, where the MP and the NEA are placed in the same slab but farther apart so that they do not interact. The interaction energy between the MP and NEA in the docking complexes has been calculated by subtracting the total energy of the reference structures from the total energy of the interacting docking complex structures.

Docking Complexes for *R*-NEA Adsorbed on Dibridge[6] Sites with pro-*R* MP

Eight reasonable starting geometries were identified for *keto* methyl pyruvate[3] adjacent to the chiral ethylamine group of *endo* NEA. The converged geometries are displayed in Supplementary Figure 6.

Interaction energies are calculated from:

$$E_{int} = E_{tot} - E_{tot}\text{ (Reference structure)} \qquad 1$$

and the energies are displayed in Supplementary Table 2.

For the combination of *keto* methyl pyruvate and *exo* NEA, six reasonable starting geometries were considered and the converged geometries are displayed in Supplementary Figure 7 and the energies are listed in Supplementary Table 3.

Combinations of syn, *enol* methyl pyruvate and NEA were also considered. For the combination of *enol* methyl pyruvate and *endo* NEA, seven reasonable starting geometries were considered and the converged geometries are displayed in Supplementary Figure 8 and the energies are listed in Supplementary Table 4. Finally, eleven reasonable starting geometries were identified for *enol* methyl pyruvate and *exo* NEA. The converged geometries of those calculations are shown in Supplementary Figure 9 and the energies are summarized in Supplementary Table 5.

Seven reasonable starting geometry were identified for anti-*enol* methyl pyruvate and *exo* NEA and the converged geometries are listed in Supplementary Figure 10 and the energies are

summarized in Supplementary Table 6. The initial calculations without van der Waals' interaction were used to exclude energetically unfavorable docking complexes and these corrections were included for the most stable geometries as the last step.

Docking Complexes for *R*-NEA Adsorbed on Dibridge[6] Sites with pro-*S* MP

Similar calculations are carried out for combinations of pro-*S* conformer of *keto*, syn and anti *enol* forms of MP (shown in Supplementary Figure 4) with the two forms of NEA adsorbed on a dibridge[6] site, initially without including van der Waals interactions and the results are summarized in this section. Supplementary Figure 11 show the calculated structures of *endo* NEA with the *keto* form of methyl pyruvate and the interaction energies are shown in Supplementary Table 7. Supplementary Figure 12 shows the calculated structures of *exo* NEA with the *keto* form of methyl pyruvate and the interaction energies are shown in Supplementary Table 8. Supplementary Figures 13, 14 and 15 show the combinations of *endo* NEA and the syn-*enol* MP, *exo* NEA and the syn-*enol* MP and *exo* NEA with the anti-*enol* MP respectively and the corresponding energies are summarized in Supplementary Tables 9, 10 and 11 respectively.

Most Stable Docking Complexes and their Equilibrium Distributions

Supplementary Table 12 summarizes the interaction energies of the most stable complexes among all the possible structures that are shown in Supplementary Figures 6 to 15.

The final most stable pro-*R* docking complexes (Supplementary Table 12) are summarized in Supplementary Figure 16 and the pro-*S* docking complexes (Supplementary Table 12) are summarized in Supplementary Figure 17 which also shows the simulated images using the Tersoff-Hamann method,[6, 7] and their relative equilibrium populations. The equilibrium populations are obtained using the calculated interaction energies assuming Boltzmann probabilities at the experimental sample temperature of 120 K and are normalized to unity.

LEED I/V Measurements

LEED intensity versus beam energy (I/V) curves for NEA-covered Pd(111) were measured using a Varian four-grid LEED system and the diffraction patterns recorded with a digital camera and the spot intensities were measured using ImageJ software.[8] The resulting I/V curve for the (1,0) beam is shown in Supplementary Figure 18.

The NEA coverages were determined by carbon monoxide site blocking as described previously,[9] and displayed in Supplementary Figure 18. Since NEA is disordered on Pd(111),[5] the I/V curves were analyzed using the substrate (1,0) beams[10, 11, 12] and by comparing the Pendry R-factors[13] for the most stable calculated geometries of *exo* and *endo* NEA on dibridge[6] and dibridge[7] sites, without allowing the structures to relax. The Pendry R factor was calculated using C-LEED software[14] by simulating the I/V curves for the calculated geometries of NEA both on dibridge[6] and dibridge[7] sites without allowing the structures to relax. The agreement between the calculated and simulated I/V curves was estimated using the Pendry R factor, where smaller R factors indicate better agreement between the simulated and measured I/V curves. The resulting values are summarized in Supplementary Table 13, where the lower R factors for NEA on the dibridge [6] site confirms that this is the favored adsorption site.

Classification of Docking Complexes

Previous work to characterize docking complexes between prochiral reactants, 2,2,2-trifluoroacetophenone (TFAP) and 3,3,3-methyltrifluoropyruvate (MTFP, the fluorinated analog of MP) defined two angles.[15] The first angle, Φ is that subtended by the long axis of the coadsorbed prochiral molecule (TFAP or MTFP) and a line between the bright spot in the TFAP or MTFP and the center of the naphthyl ring in NEA. The second angle, Θ is that subtended between the long axis of the naphthyl ring in NEA and a line between the bright spot in the TFAP or MTFP and the

center of the naphthyl ring. STM image contrast in TFAP and MTFP was provided by the presence of fluorines in the molecule, which were not present in MP, so that docking complexes were identified from their STM images only from the angle subtended between the long axis of the MP molecules and the long axis of NEA as illustrated in Supplementary Figure 19. The angle is defined as that subtended between the long axes of ellipses superimposed over the naphthyl group of NEA and a co-adsorbed MP. To avoid human bias, the angles were initially classified independently by two people and only those complexes on which there was agreement between the two were included in the distributions. A total of 103 clearly identifiable docking complexes were included and the sampling error *S* was calculated from $\sqrt{\frac{p(1-p)}{n}}$, where *p* is the probability of occurrence of a docking complex and *n* is the number of samples (103). The corresponding errors in the equilibrium distributions were estimated by calculating the probability distribution at 130 and 110 K to account for possible temperatures differences between STM imaging experiments.

Factors Controlling Docking Complex Formation

Supplementary Figure 20 shows that the interaction energies of docking complexes between MP and *R*-NEA (■) depend on the C=O····H_2N hydrogen-bonding distance such that docking complexes with hydrogen-bonding distances less than ~0.18 or greater than ~0.25 nm comprise less than 1% of the observed complexes. A line is shown as a guide to the eye and reveals that the strongest interaction occurs at a C=O····H_2N distance of 0.22 ± 0.01 nm, in good agreement with the optimum distances calculated for C=O····H_2N hydrogen bonds.[16]

The red points on this curve deviate from the trend and are for Pro-*S* MP docking with *R*-NEA (Figure 17F and 17G). In order to obtain insights into the difference, the docking complex in Supplementary Figure 17F (for Pro-*S* MP) is compared with the Pro-*R* structure in

Supplementary Figure 16G and the complex in Supplementary Figure 17G (for Pro-*S* MP) is compared with the Pro-*R* structure in Supplementary Figure 16H. These docking complexes are compared since the NEA and MP adsorbate locations are identical and have similar C=O····H_2N hydrogen-bonding distances. Comparison of the structures reveal that the distances between the NEA NH_2 group and the OCH_3 and OH groups on MP differ between the Pro-*S* and Pro-*R* structures, expected to give rise to different dipole-dipole interactions for the Pro-*S* and Pro-*R* forms of MP and NEA. The dipole-dipole interactions are estimated using typical dipole moments for the OCH_3 (~2.9 D), OH (~1.7 D) and NH_2 (~1.2 D) groups,[17] and the dipole-dipole interaction energies are calculated from:

$$E_{int} = \frac{\mu_1 \mu_2}{4\pi\epsilon_0 r_{12}^3}(\cos\theta_{12} - 3cos\theta_1 cos\theta_2) \qquad 2$$

where μ_1 and μ_2 are the dipole moments of the interacting dipoles separated by a distance r_{12}. ϵ_0 is the permittivity of free space, θ_{12} is the angle between the vectors along dipoles *1* and *2*, θ_1 and θ_2 are the angles subtended between dipoles *1* and *2* and a vectors (of length r_{12}) between the dipoles. The results are summarized in Supplementary Table 14.

Supplementary References

1. Lenz DH, Conner Jr WC. Computer analysis of the cracking patterns of deuterated hydrocarbons. *Anal Chim Acta* **173**, 227-238 (1985).

2. Burkholder L, Garvey M, Weinert M, Tysoe WT. Structure of methyl pyruvate and α-(1-naphthyl)ethylamine on Pd(111). *J Phys Chem C* **115**, 8790-8797 (2011).

3. Garvey M, Bai Y, Boscoboinik JA, Burkholder L, Sorensen TE, Tysoe WT. Identifying molecular species on surfaces by scanning tunneling microscopy: Methyl pyruvate on Pd(111). *J Phys Chem C* **117**, 4505-4514 (2013).

4. Demers-Carpentier V, *et al.* Direct observation of molecular preorganization for chirality transfer on a catalyst surface. *Science* **334**, 776-780 (2011).

5. Boscoboinik JA, Bai Y, Burkholder L, Tysoe WT. Structure and distribution of S-α-(1-naphthyl)-ethylamine on Pd(111). *J Phys Chem C* **115**, 16488-16494 (2011).

6. Tersoff J, Hamann DR. Theory and application for the scanning tunneling microscope. *Phys Rev Lett* **50**, 1998-2001 (1983).

7. Tersoff J, Hamann DR. Theory of the scanning tunneling microscope. *Phys Rev B* **31**, 805-813 (1985).

8. Schneider CA, Rasband WS, Eliceiri KW. NIH Image to ImageJ: 25 years of image analysis. *Nat Meth* **9**, 671-675 (2012).

9. Burkholder L, Stacchiola D, Boscoboinik JA, Tysoe WT. Enantioselective chemisorption on model chirally modified surfaces: 2-Butanol on α-(1-naphthyl)ethylamine/Pd(111). *J Phys Chem C* **113**, 13877-13885 (2009).

10. Zheng T, Tysoe WT, Poon HC, Saldin DK. Structure determination of ordered and disordered organic molecules on a surface from the substrate diffraction spots in low energy electron diffraction: $(\sqrt{3} \times \sqrt{3})R30^{o}$-$C_2H_2$ and disordered CH_3OH on Pd(111). *Surf Sci* **543**, 19-28 (2003).

11. Poon HC, Weinert M, Saldin DK, Stacchiola D, Zheng T, Tysoe WT. Structure determination of disordered organic molecules on surfaces from the Bragg spots of low-energy electron diffraction and total energy calculations. *Phys Rev B* **69**, (2004).

12. Zheng T, Stacchiola D, Poon HC, Saldin DK, Tysoe WT. Determination of the structure of disordered overlayers of ethylene on clean and hydrogen-covered Pd(111) by low-energy electron diffraction. *Surf Sci* **564**, 71-78 (2004).

13. Pendry JB. Reliability factors for LEED calculations. *J Phys C: Solid State* **13**, 937 (1980).

14. Held G, Bessent MP, Titmuss S, King DA. Realistic molecular distortions and strong substrate buckling induced by the chemisorption of benzene on Ni{111}. *J Chem Phys* **105**, 11305-11312 (1996).

15. Groves MN, *et al.* Structure determination of chemisorbed chirality transfer complexes: Accelerated STM analysis and exchange-correlation functional sensitivity. *Surf Sci* **629**, 48-56 (2014).

16. Lu N, Chung W-C, Ley RM, Lin K-Y, Francisco JS, Negishi E-I. Molecularly tuning the radicaloid N–H···O═C hydrogen bond. *J Phys Chem A* **120**, 1307-1315 (2016).

17. Minkin VI, Osipov OA, Zhdanov IA. *Dipole moments in organic chemistry*. Plenum Press (1970).
